# Fetal hypoxia results in sex- and cell type-specific alterations in neonatal transcription in rat oligodendrocyte precursor cells, microglia, neurons, and oligodendrocytes

**DOI:** 10.1186/s13578-023-01012-8

**Published:** 2023-03-17

**Authors:** Isaac Kremsky, Qingyi Ma, Bo Li, Chiranjib Dasgupta, Xin Chen, Samir Ali, Shawnee Angeloni, Charles Wang, Lubo Zhang

**Affiliations:** 1 Department of Basic Sciences, Loma Linda University School of Medicine, Loma Linda, CA USA; 2 Center for Genomics, Loma Linda University School of Medicine, Loma Linda, CA USA; 3 Lawrence D. Longo MD Center for Perinatal Biology, Department of Basic Sciences, Loma Linda University School of Medicine, Loma Linda, CA 92350 USA

**Keywords:** Fetal hypoxia, Rat brain RNA-seq, NF kappa B

## Abstract

**Background:**

Fetal hypoxia causes vital, systemic, developmental malformations in the fetus, particularly in the brain, and increases the risk of diseases in later life. We previously demonstrated that fetal hypoxia exposure increases the susceptibility of the neonatal brain to hypoxic-ischemic insult. Herein, we investigate the effect of fetal hypoxia on programming of cell-specific transcriptomes in the brain of neonatal rats.

**Results:**

We obtained RNA sequencing (RNA-seq) data from neurons, microglia, oligodendrocytes, A2B5^+^ oligodendrocyte precursor cells, and astrocytes from male and female neonatal rats subjected either to fetal hypoxia or control conditions. Substantial transcriptomic responses to fetal hypoxia occurred in neurons, microglia, oligodendrocytes, and A2B5^+^ cells. Not only were the transcriptomic responses unique to each cell type, but they also occurred with a great deal of sexual dimorphism. We validated differential expression of several genes related to inflammation and cell death by Real-time Quantitative Polymerase Chain Reaction (qRT-PCR). Pathway and transcription factor motif analyses suggested that the NF-kappa B (NFκB) signaling pathway was enriched in the neonatal male brain due to fetal hypoxia, and we verified this result by transcription factor assay of NFκB-p65 in whole brain.

**Conclusions:**

Our study reveals a significant impact of fetal hypoxia on the transcriptomes of neonatal brains in a cell-specific and sex-dependent manner, and provides mechanistic insights that may help explain the development of hypoxic-ischemic sensitive phenotypes in the neonatal brain.

**Supplementary Information:**

The online version contains supplementary material available at 10.1186/s13578-023-01012-8.

## Background

Large clinical and preclinical studies have revealed that an adverse fetal environment increases the risk of cardiovascular, metabolic, and neurobehavioral diseases; these observations have led to what is known as the “fetal origins of adult disease” hypothesis [1–4]. Fetal hypoxia is a common form of prenatal stress that can occur under a variety of conditions, including pregnancy at high altitude, maternal smoking, maternal anemia, placental insufficiency, and cord compression. Fetal hypoxia can cause developmental malformations, particularly in the brain [5–8]. This can lead to multiple neurodevelopmental disorders in offspring, such as cerebral palsy, autism, and cognitive dysfunction [8, 9]. Further, our previous studies in rodents demonstrate that fetal hypoxia promotes the development of neurological disease-sensitive phenotypes in offspring, including neonatal hypoxic-ischemic encephalopathy, ischemic stroke, and age-related cognitive decline [10–13].

Due to the protracted nature of brain development, the time window of fetal hypoxia exposure is a critical factor that affects the ultimate consequences of fetal hypoxia. The processes at each developmental stage affect a specific set of developmental processes in the brain, and interfering with each of these can have a specific set of consequences on specific cell types. Neurons (NRs) are the dominant cell type and basic functional unit in the brain; a series of events related to NR growth proceed during the perinatal period, such as neurogenesis and synaptogenesis, to form the mature nervous system [14, 15]. Glial cells, including microglia (MGs), astrocytes (ACs), and oligodendrocytes (ODCs), comprise at least 50% of all cells in the brain [16, 17]. The prenatal period is a critical stage for glial proliferation, maturation and plasticity. For example, the second wave of ODC progenitor cell specification and migration to the central nervous system (CNS) occurs at this time [18–20]. The migration of MGs into the brain happens around the mid-gestation stage (E9) in mouse, and MGs start to achieve ramified morphology and distribute in the cerebral cortex prenatally [21, 22]. Astrogenesis initiates at the end of gestation (E18) and lasts until neonatal day 7 in mice [23]. The interaction of glial cells with NRs is also indispensable for neurodevelopment and later physiological maintenance. Mounting evidence suggests that prenatal hypoxia affects the number of NRs, synaptic density, MG morphology and function, AC differentiation and migration, and ODC maturation [24–26].

Although the deleterious effect of fetal hypoxia on neurodevelopment and progression of neurological disorders in later life have been phenotypically characterized, the underlying cellular and molecular mechanisms remain unclear. Several mechanisms have been proposed, however, including increased apoptotic cell death and loss of stem or progenitor cells due to energy deprivation [27], increased reactive oxygen species (ROS) and reactive nitrogen species (RNS), and maturation stage-specific vulnerability of ODC progenitors in the periventricular leukomalacia region [28, 29]. Hypoxia-induced fetal brain injury is also associated with an inflammatory response via overexpression of pro-inflammatory cytokines and pro-apoptotic proteins [9, 30].

Male infants are known to have a larger inflammatory response to ischemic insults than females [31], and fetal hypoxia has been observed to have a sexually dimorphic effect on the mitochondria of fetal guinea pigs [32]. The precise mechanisms that give rise to sexually dimorphic outcomes, and their precise relationship to more general, sex-independent outcomes, are unknown. Understanding the nature of sexually dimorphic responses to fetal hypoxia therefore has the potential to help understand the precise mechanisms of action of fetal hypoxia.

In order to gain insights into the specific brain cell types affected by fetal hypoxia, as well as the degree of sexual dimorphism, we performed next-generation RNA sequencing (RNA-seq) of multiple brain cell types in neonatal rats subjected to either fetal hypoxia or normoxia (controls): NRs, MGs, ACs, ODCs, and ODC precursor cells (A2B5^+^). We observed that fetal hypoxia induced substantial transcriptomic changes in the neonatal brain in a cell-type specific and sex-dependent manner in NRs, MGs, ODCs, and A2B5^+^ cells. With pathway and transcription factor motif analyses, we found that immune- and inflammation-related genes were highly enriched in each cell type. A functional assay revealed that the NF-kappa B (NFκB) signaling pathway is specifically activated in the male brain. Thus, this study demonstrates that fetal hypoxia has unique effects on neonatal transcription across multiple brain cell types, and that many of these alterations are sexually dimorphic. The diversity of cell- and sex-specific responses to fetal hypoxia may underly the diversity of disease outcomes that can occur after fetal hypoxia exposure.

## Results

### Sex- and cell type-dependent transcriptional changes in the neonatal rat brain due to fetal hypoxia

First, we measured the brain and body masses of neonatal rats subjected to fetal hypoxia versus controls. Both brain and body masses were decreased by fetal hypoxia, but the brain to body mass ratio was increased (Additional file 1: Fig. S1A), confirming a typical asymmetric growth restriction resulting from fetal hypoxia [12, 13].

We therefore performed RNA-seq on A2B5^+^ cells, ODCs, ACs, NRs, and MGs in both male and female rat pups subject to fetal hypoxia or control conditions. Four biological replicates from each treatment group were sequenced, for a total of 80 RNA-seq samples. Prior to sequencing, all RNA samples were subject to and passed quality control measures (Additional file 1: Fig. S1B). We sequenced to an average depth of 24.3 million reads per sample, across all samples. All samples showed uniformly high sequencing quality across both read pairs and passed the FastQC [33] quality control measures (Additional file 1: Fig. S1C). Fragments per kilobase per million (FPKM) normalized values of gene expression were highly correlated across replicates within the same treatment group (Additional file 1: Fig. S1D).

**Fig. 1:**
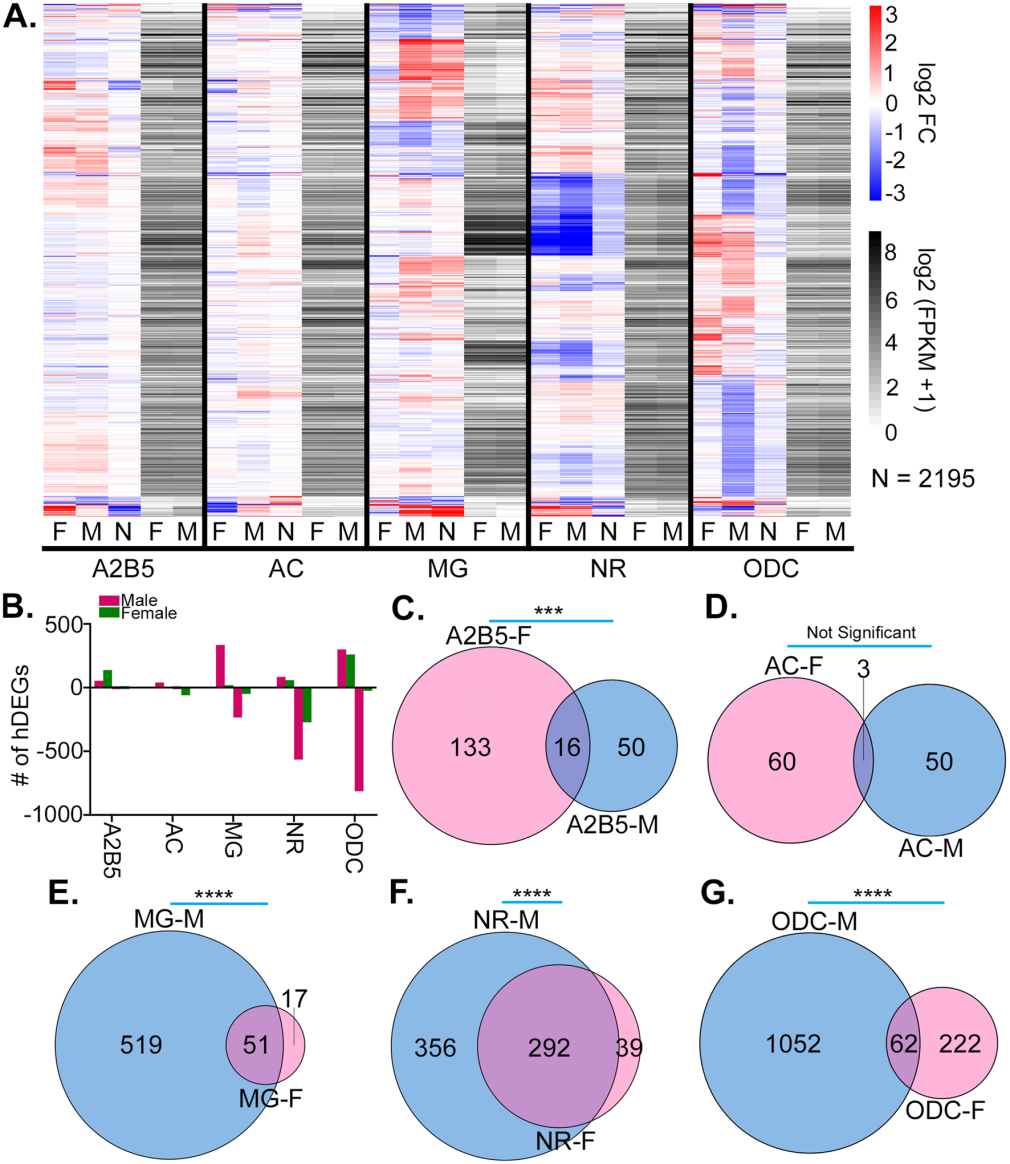
Unique and sexually dimorphic transcriptomic changes in the neonatal brain due to fetal hypoxia. **A**. Heatmap of log_2_ Fold Change (FC) values of hDEGs from the indicated samples, along with normalized gene expression (log_2_ (FPKM + 1)) of male and female normoxic controls. Genes are ordered by hierarchical clustering of the log_2_ FC values. In the log_2_ FC columns (red-blue), M indicates hypoxia vs. control in males, F indicates hypoxia vs. control in females, and N indicates male vs. female in normoxic controls. In the gene expression columns (white-black), M indicates the pooled gene expression of normoxic males, and F indicates the pooled gene expression of normoxic females. **B**. Bar plots of the number of hDEGs in samples of the indicated cell type and sex. Bars on the positive y-axis indicate the number of DEGs upregulated due to fetal hypoxia, while bars on the negative y-axis indicate the number of DEGs downregulated due to fetal hypoxia. **C–G**. Venn diagrams showing the number of hDEGs distinct and overlapping between male (M) and female (F). P-values displayed are for the null hypothesis that the number of male and female hDEGs are the same, by Fisher’s Exact Test. P-value cutoffs are as follows: * p < .01; ** p < .001; *** p < 10^–5^; **** p < 10^–10^

We then determined the set of differentially expressed genes (DEGs) of male vs. female, as well as fetal hypoxia treatment versus controls. 2,195 fetal hypoxia-induced DEGs (hDEGs) were identified, with the largest number of hDEGs occurring in ODCs, NRs, and MGs (Fig. 1A). In terms of the number of hDEGs, the cell type that was most affected was male ODC, which had 1,114 hDEGs (Additional file 2: Table S1). Interestingly, fetal hypoxia affected the expression of more genes than were altered by sex (Additional file 2: Table S2). A small number of genes showed interaction effects, whereby changes in both sex and hypoxia treatment together affected gene expression more than the sum of the individual effects (Additional file 2: Table S3). Strikingly, the number of hDEGs was highly sex-dependent in all brain cell types except ACs (Figs. 1B–G). While some of these sex-specific hDEGs were sexually dimorphic in normoxic control animals, many were not (Fig. 1A). All cell types contained sex-dependant hDEGs that weren’t sexually dimorphic in normoxic controls, with the largest number of these occurring in ODCs. As an example, Nfe2l2 expression was affected by hypoxia to a much greater extent in ODC males than females, and had similar expression in male and female controls (Additional file 1: Fig. S1E).

Considering all hDEGs equally as above does not provide any information about which genes have the largest changes in absolute expression and therefore could have a larger influence on certain phenotypes. We therefore performed Principal Component Analysis (PCA) on the RNA-seq data in a way that weights genes with high expression variability more heavily than those with low expression variability (see Methods). When including all samples in a single PCA plot, glial cells (MG, AC, and ODC) clustered closely together and were far from NRs (Additional file 1: Fig. S1F), consistent with the fact that glial cells are more similar to each other than to NRs in terms of neonatal phenotypes and function. The effect of sex and hypoxia on the PCA were both small relative to differences between cell types, again consistent with expectation, since large transcriptome variation across cell types is well known, whereas the number of hDEGs is small relative to the transcriptome sizes of the cells. Our PCA is therefore capable of recapitulating large-scale phenotypic differences between samples.

**Fig. 2:**
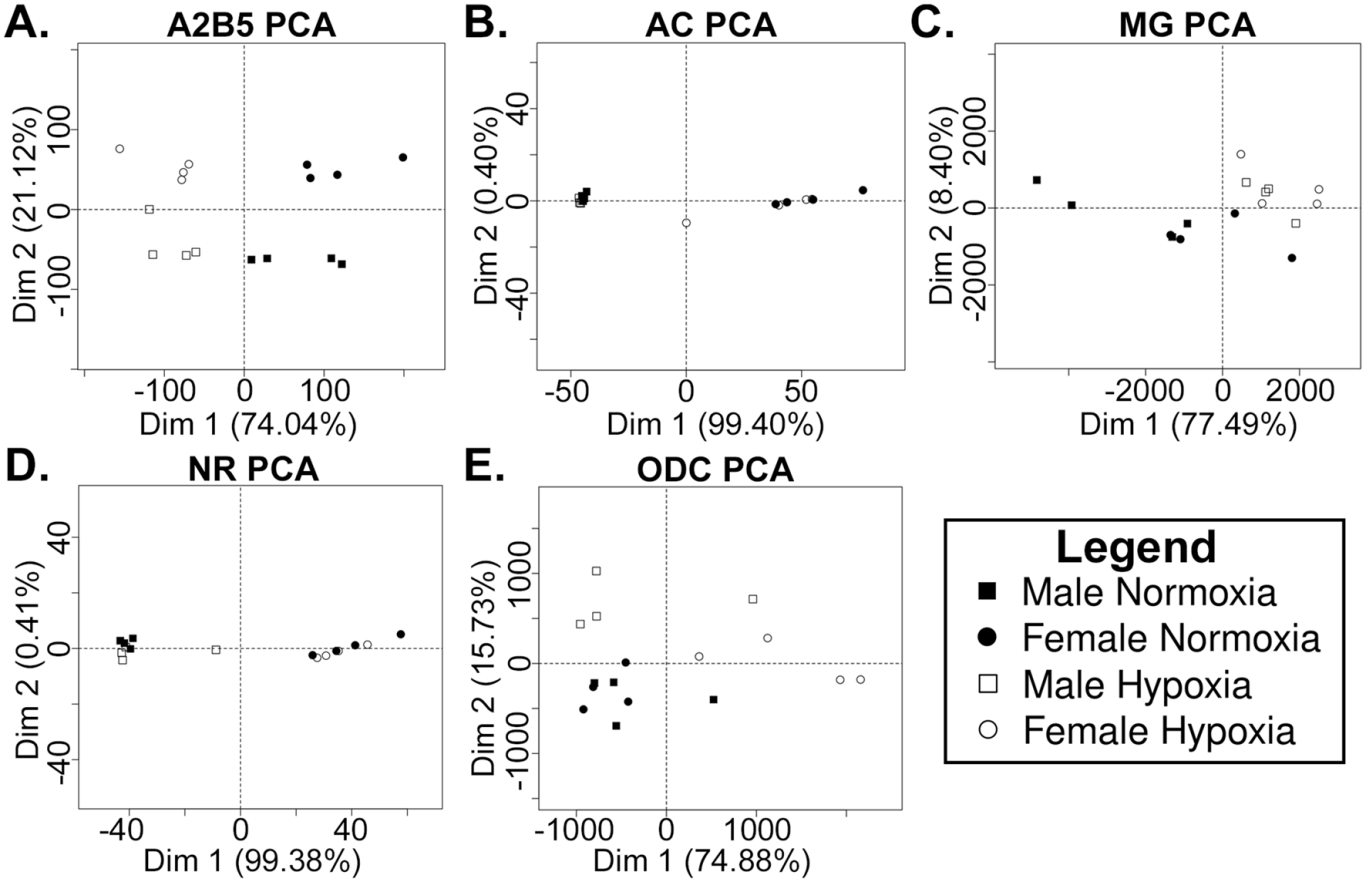
Principal component analysis of RNA-seq data. The first two principal components in cell type specific PCAs for **A**. A2B5 + cells; **B**. astrocytes; **C**. microglia; **D**. neurons; **E**. oligodendrocytes

Given that the results of our global PCA analysis were consistent with expected overall phenotypic variation, which is primarily driven by differences in cell type transcriptomes, we performed a PCA on the samples of each cell type separately in order to more clearly assess the effects of sex and fetal hypoxia on highly changed transcripts. Interestingly, and consistent with the relative number of hDEGs in each sample (Fig. 1), the relative strength and degree of sexual dimorphism of the hypoxia effect was unique to each cell type. Based on PCA, hypoxia was the major effector in A2B5^+^ cells (Fig. 2A and Additional file 1: Fig. S2A). Although there was a significant difference in the number of hDEGs between A2B5^+^ males and females (Fig. 1C), the PCA of A2B5^+^ cells suggests a much smaller degree of sexual dimorphism in genes with the highest variability in A2B5^+^ cells. On the other hand, hypoxia only had a weak effect in ACs when compared to sex differences in the PCA (Fig. 2B and Additional file 1: Fig. S2B), consistent with the relatively low number of AC hDEGs (Fig. 1B). MGs were substantially affected by hypoxia, but the effect on the PCA was stronger on average in males than in females (Fig. 2C and Additional file 1: Fig. S2C), consistent with a greater number of MG hDEGs in males than females (Fig. 1E). A weak effect of hypoxia was evident in the PCA for NRs (Fig. 2D and Additional file 1: Fig. S2D) despite a large number of hDEGs in NRs (Fig. 1F), suggesting that in NRs, hypoxia induces relatively small changes in absolute gene expression as compared to sex. In the PCA for ODCs, hypoxia had a larger effect on females on average than males (Fig. 2E and Additional file 1: Fig. S2E) despite the fact that there were more hDEGs in males than females (Fig. 1G). Thus, highly changed transcripts are affected more in female ODCs than males, despite there being many more male than female hDEGs. Interestingly, the sex difference in normoxic MGs was largely ablated by fetal hypoxia, suggesting that, for transcripts with the largest changes in MG, the sexual dimorphism is driven by the convergence of sexually distinct normal phenotypes to a common hypoxia phenotype. On the other hand, the PCA of ODCs suggests that under normal conditions, the most highly variable ODC genes are expressed similarly in males and females, but these genes respond to fetal hypoxia uniquely in each sex.

Taken together, these results demonstrate that fetal hypoxia induces unique and sex-dependent transcriptomic alterations in neonatal A2B5^+^ cells, MGs, NRs, and ODCs, and suggest that these transcriptomic alterations may result in significant and sexually dimorphic alterations in the phenotypes of each of these cell types.

### Pathway analysis of the hDEGs

**Fig. 3:**
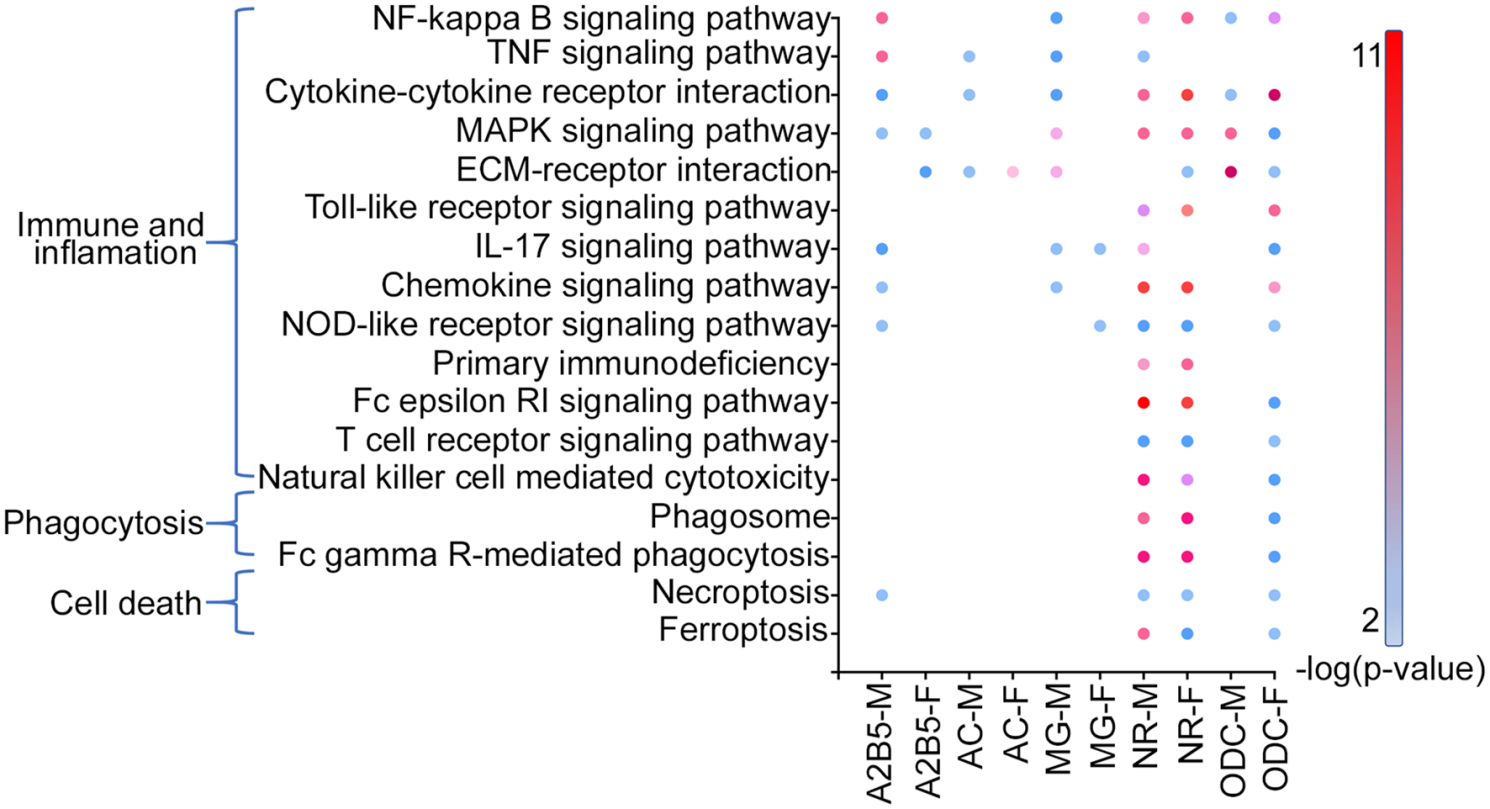
Enriched pathways from fetal hypoxia induced DEGs. Plot indicating the significantly enriched pathways (P < .05) in each of the cell types and sexes indicated

To get insights into the biological consequences of fetal hypoxia on the neonatal brain, we performed pathway analyses on the hDEGs independently for each cell type and sex. Each cell type and sex had a unique set of enriched pathways (Fig. 3). Phagocytosis and cell death were primarily enriched in NRs and ODCs, while all cell types had enrichment of at least one pathway related to immunity and inflammation. For example, the NF-kappa B signaling pathway was significantly enriched in male A2B5^+^ cells and MGs, and both male and female NRs and ODCs. Transcriptional changes in both the canonical and non-canonical NF-kappa B pathways occurred in male MGs (Additional file 1: Fig. S3A). Diagrams of additional pathways are included for reference (Additional file 1: Fig. S3B–K).

### Transcription factor binding motifs associated with hDEGs in brain cells

**Fig. 4:**
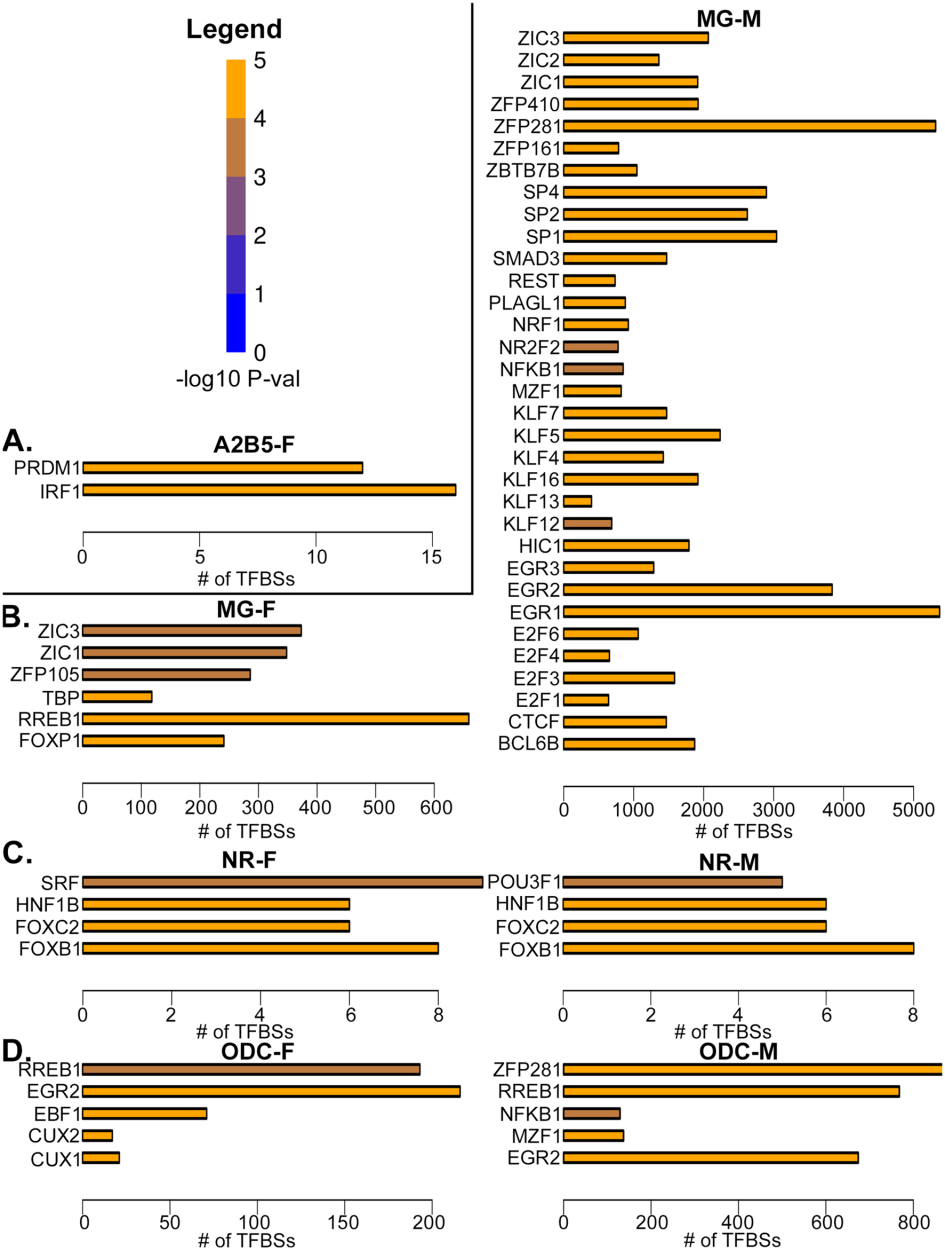
Enriched TF binding motifs near fetal hypoxia-induced, neonatal DEGs. Bar plots indicating the number of putative TF binding sites (TFBSs) at enhancers, based on sequence motif hits near fetal hypoxia DEGs in **A**. A2B5 + cells; **B**. microglia; **C**. neurons; **D**. oligodendrocytes. Indicated P-values are by Fisher’s Exact Test

An important driver of environmentally-induced changes in gene expression is changes in transcription factor (TF) binding at enhancers. We therefore performed a motif enrichment analysis at enhancers near hDEGs for all brain cell types that had enhancer annotations available in the rat (Fig. 4A–D). Only TFs that are expressed according to the RNA-seq data for the given cell type and sex were shown. Male MGs manifested the highest number of hypoxia-enriched, putative TF binding sites (TFBSs). Examples illustrating the expression of some of these TFs, and changes in gene expression of a few example genes with nearby TF motifs are shown in Additional file 1: Fig. S4A–L. Interestingly, the NF Kappa B1 subunit (NFκB1) motif was significantly enriched in male MGs and ODCs, consistent with the enrichment of the NF Kappa B pathway amongst hDEGs in both cell types (Fig. 3).

### Validation of fetal hypoxia-induced DE of genes related to inflammation and cell death by qRT-PCR

**Fig. 5:**
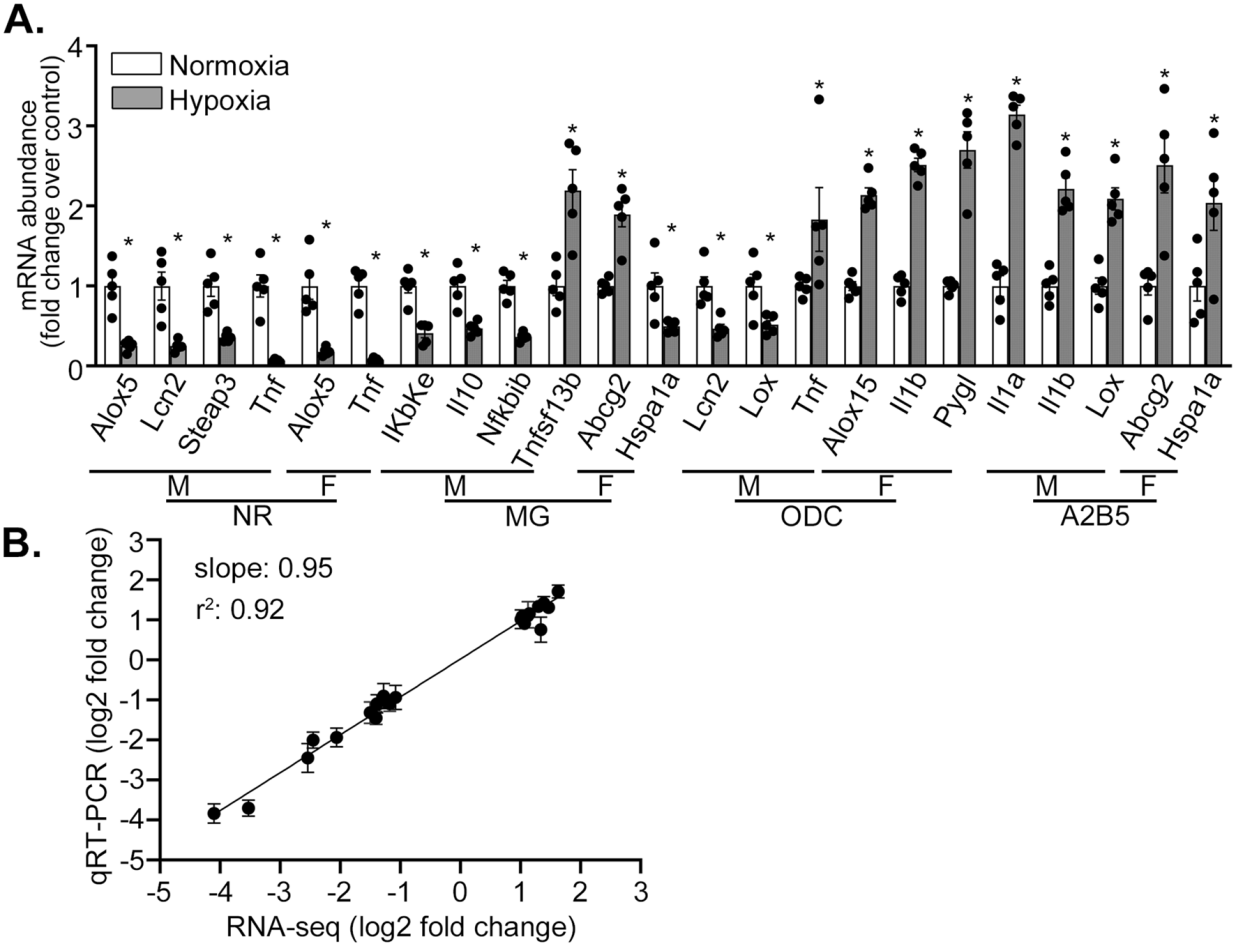
Validation of inflammation and cell death related fetal hypoxia-induced, neonatal DEGs. **A**. Quantification of mRNA abundance of genes by qRT-PCR in the indicated cell types sorted from neonatal brains with fetal hypoxia or normoxia exposure. NR, neurons; MG, microglia; ODC, oligodendrocytes; A2B5, A2B5 + cells; M, male; F, female. Error bars indicate mean ± SEM. P-values are by t-test, with n = 5 pups per group. *P < .05. **B**. Comparison of hypoxia-induced log_2_ Fold Changes according to RNA-seq vs. qRT-PCR of hDEGs validated by qRT-PCR

The results above based on RNA-seq demonstrate putative alterations in a number of genes involved in both inflammation and cell death. To validate this, we performed qRT-PCR to confirm select DEGs in male and female MGs, NRs, ODCs, and A2B5^+^ cells. The following hDEGs were validated in the indicated cell types: *Il10, Tnfsf13b, Nfkbib,* and *Ikbke* in male MGs; *Abcg2* and *Hspa1a* in female MGs; *Alox5, Steap3, Tnf,* and *Lcn2* in male NRs; *Alox5* and *Tnf* in female NRs; *Tnf, Lox,* and *Lcn2* in male ODCs; *Il1b* and *Alox15* in female ODCs; *Il1a, Il1b,* and *Lox* in male A2B5^+^ cells; *Abcg2* and *Hspa1a* in female A2B5^+^ cells (Fig. 5A). The qRT-PCR results were strongly correlated to the corresponding results in RNA-seq (Fig. 5B), indicating a strong concordance between the DEG results of RNA-seq and of qRT-PCR analysis.

### Fetal hypoxia enhanced the activity of NFκB-p65 in the brain

**Fig. 6:**
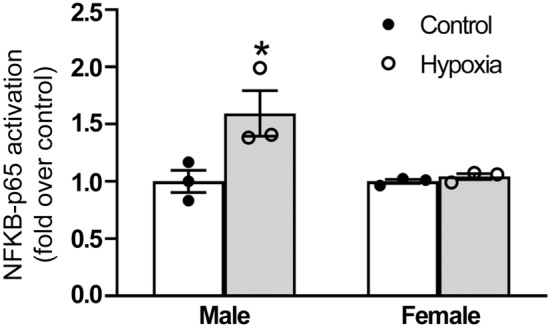
Effect of fetal hypoxia on NFκB-p65 activity in the brains of neonatal rats. Plots showing the average (tops of bars), standard error (error bars), and distribution (individual points) of the fetal hypoxia-induced increase of NFκB-p65 nuclear translocation in whole brains of the indicated samples. P-values are determined by two-way ANOVA. *P < 0.05, hypoxia versus control

To validate the activation of the NFκB pathway due to fetal hypoxia in the neonatal rat brain, we conducted a protein assay of nuclear translocation of a key NFκB subunit, p65, in the neonatal brain. Importantly, we found that NFκB-p65 activity was significantly increased by fetal hypoxia in male, but not female, neonatal brains (Fig. 6).

## Discussion

In this study, we used RNA-seq of sorted brain cells of rat pups to analyze neonatal transcriptomic changes associated with fetal hypoxia. To the best of our knowledge, this is the first study to measure the transcriptional profile of cell populations in the developing brain of rats using RNA-seq. The results indicate that fetal hypoxia induces substantial transcriptomic alterations in neonatal NRs and glial cells, in a cell type- and sex-specific manner.

Our previous studies demonstrate that fetal hypoxia exposure increases the susceptibility of the neonatal brain to hypoxic-ischemic insult [12, 34], which mainly occurs during the perinatal period in humans. Brain development of humans at birth is roughly equivalent to rodents at postnatal days 7–10 [35–39]. The main goal of this study was to explore the transcriptomic response of brain cell populations with hypoxic-ischemic sensitive phenotypes, so we sampled rat brains at postnatal day 7. Due to the heterogeneity of brain development and the ability of self-repair or -renewal, the transcriptomic response of each brain cell type may be different at different life stages following hypoxia exposure in the fetal stage. For example, our previous study determined that gestational hypoxia significantly increases the expression of genes related to astrogliosis and microgliosis, but doesn`t affect the expression of synaptic proteins, in the brain of 2-month-old mouse offspring [13].

Our pathway analyses suggest that immune and inflammatory pathways may be highly affected by fetal hypoxia in neonatal MGs, NRs, and ODCs. This could contribute to the activation of cell death pathways in NRs and ODCs, thus leading to increased susceptibility of the brain to later insults. In addition, we demonstrated an overall reduction in neonatal brain mass due to fetal hypoxia. An increase in inflammation and cell death could be the cause of this reduction in brain mass, although further studies are needed to directly link the two. We performed qRT-PCR in MGs, NRs, ODCs, and A2B5^+^ cells, and directly demonstrated that fetal hypoxia induces neonatal transcriptomic changes in genes with known involvement in inflammatory and cell death pathways.

Among the pathways and TF motifs we analyzed bioinformatically, NFκB signaling was commonly enriched in multiple brain cell types, and this culminated in verification of an increased nuclear translocation and activity of NFκB-p65 in hypoxia-exposed male brains. Sexually dimorphic responses to fetal hypoxia have been observed in the past, but the precise mechanisms are unknown. The finding that NFκB activity is increased in the neonatal brain in a sex-dependent manner may provide insight into the mechanisms involved in sexually dimorphic responses to fetal hypoxia. The NFκB signaling pathway plays a key role in inflammation by regulating immune response-related gene expression.

Our recent study showed that NFκB-p65 activation increases the production of an important inflammatory response gene, IL-1β, after neonatal hypoxic-ischemic brain injury in mice [40]. Further, it has been reported that fetal stress causes priming of MG activation [41–43], which is regulated by the NFκB signaling pathway [44]. We validated immune response related hDEGs in male MGs using qRT-PCR and found that *Il10, Ikbke, and Nfkbib* were downregulated and *Tnfsf13b* was upregulated. *Il10* exerts anti-inflammatory actions in glial cells and can inhibit activation of the NFκB pathway [45, 46]. *Nfkbib* and *Ikbke* encode proteins of NFκB endogenous inhibitors [47]. *Tnfsf13b* is a member of the TNF superfamily, which is a sensome gene of MGs [48], and is active, along with its receptor, in inflammation [49]. Thus the results of our study suggest that fetal hypoxia may prime male MGs through the activation of the NFκB signaling pathway.

Interestingly, immune and inflammation pathways were also activated by fetal hypoxia in ODCs and A2B5^+^ cells. For example, the key cytokines *Il1b* and *Il1a* were upregulated in the ODC lineage, which can result in excessive inflammation and ODC and A2B5^+^ cell death [50]. IL-1β is consistently upregulated by perinatal encephalopathy in both humans and experimental models [51–53], and inhibition of IL-1β reduces ODC loss following prenatal inflammation in near-term fetal sheep [54]. These findings suggest that increased inflammation after hypoxia exposure is associated with a greater risk of white matter injury after birth. As a pleiotropic cytokine, IL-1β can induce multiple effects in the injured brain. In additional to its detrimental effect, IL-1β also participates in CNS repair in the adult brain after brain injury [55]. Moreover, IL-1β regulates ODC development by blocking proliferation at the late progenitor/pro-ODC (O4^+^) stage, but does not affect proliferation of early progenitors (A2B5^+^) [56]**,** suggesting that the upregulation of IL-1β and other immune-related pathways in A2B5^+^ cells may be a tissue repair mechanism to rescue fetal stress-induced ODC death. Our pathway analysis also showed that cell death pathways were enriched in NRs and ODCs, but not A2B5^+^ cells. Therefore, the potential double-faced actions of pro-inflammatory cytokines in ODC and A2B5^+^ cells are worth further investigation.

Phagocytosis and cell death pathways were enriched in NRs and ODCs after fetal hypoxia, which may also be ascribed to immune and inflammation responses. Phagocytic NRs have been observed, with proposed involvements in debris clearance, neuronal death, and cell-to-cell spreading of disease [57]. It is still unclear which types of hypoxia-induced cell death occur in early neonatal brains. For the first time, we identified necroptosis and ferroptosis as cell death pathways that may be upregulated in neonatal NRs and ODCs exposed to fetal hypoxia. Necroptosis is unregulated necrotic death, stimulated by secretion of cytokines/chemokines, resulting in inflammation, which is initiated by TNF superfamily receptors, toll-like receptors, and interferon receptors [58, 59]. All these pathways were enriched in neonatal NRs and ODCs by RNA-seq pathway enrichment analysis.

Ferroptosis is another cell death pathway enriched in NRs and ODCs, and is characterized by a large amount of iron accumulation and lipid ROS during the cell death process [60]. In the present study, necroptosis and ferroptosis pathway alterations were supported by qRT-PCR of *Tnf*, *Steap3, Alox5*, and *Lcn2* in NRs, and *Il1β, Alox15, and Pygl* in ODCs. *Steap3* is a ferroptosis-related gene encoding an iron transporter, and dysregulation of which leads to impaired iron homeostasis and neurodegeneration [61]. Neuronal derived lipocalin-2 (*Lcn2*), another hDEG in NR, is a signature of several CNS anomalies and participates in inflammation, NR death and neurodegeneration [62–65]. *Alox5* and *Alox15* were also hDEGs in NR; these genes regulate lipid peroxidation and cause ferroptosis and inflammation [66, 67]. Moreover, Alox15 expression is increased in the brains of periventricular leukomalacia (PVL) patients [68]. The upregulation of cell death-related genes in ODCs agrees with previous findings that fetal hypoxia induces ODC loss and white matter injury. On the other hand, cell death-related genes in NRs, such as *Tnf, Lcn2* and *Alox5,* were downregulated, which has been reported to be associated with anti-inflammation and neuroprotection [65, 69, 70]. This result may suggest a recovery process in neuronal cells in the early neonatal stage from prenatal hypoxia exposure.

The proper development and function of the CNS is particularly sensitive to oxygen concentration, and hypoxia is associated with developmental malfunctions in offspring. The impact of fetal hypoxia on the neonatal brain at the cellular level is tightly regulated by transcriptomic changes at the molecular level. Our data suggest that ODCs have the greatest transcriptomic response to fetal hypoxia, followed by NRs, MGs, and A2B5^+^ cells. These data indicate that the oligodendroglial lineage is highly vulnerable to fetal hypoxia in the developing brain. Our findings are in line with the notion that PVL is mainly caused by the dysregulation of the oligodendroglial lineage in perinatal white matter [71–73]. Along with white matter injury, cortical and subcortical grey matter is also significantly affected by fetal hypoxia, resulting in neuronal damage in the premature brain [27, 74]. Indeed, fetal hypoxia can result in both subcortical white matter and gray matter injury in preterm sheep [75–77]. PVL white matter lesions are usually surrounded by astrogliosis and microgliosis [78]. The perinatal period is a critical window for microglia distribution, ramification, and maturation in the brain. Thus, MGs during this period are particularly sensitive to environmental influences, which may result in long-term changes in MG cell number and/or phenotypes [13, 79]. We have previously demonstrated that fetal hypoxia increases microgliosis and MG activation in adult mouse offspring [13].

AC was the cell type with the smallest number of hDEGs. ACs originate from neural cells at the end of the neurogenic process (E18) in rodents. Thus, a possible explanation for this is that fetal hypoxia may affect neural differentiation into ACs, leading to a reduced ratio of NRs to ACs in the immature brain [25, 80], instead of a direct effect on the AC transcriptome. Indeed, our recent study showed that prenatal hypoxia exposure significantly increases astrogliosis in the brain of adult offspring [13]. More research is needed to understand the mechanistic alterations of neural differentiation in response to fetal hypoxia.

## Conclusions

In the present study, we performed RNA-seq on sorted brain cells to analyze transcriptomic changes in rat pups after fetal hypoxia exposure. We generated global gene expression profiles for five major brain cell populations: NRs, MGs, ODCs, ACs and A2B5^+^ cells. Our data indicate that immune- and inflammation-related pathways are activated in neonatal NRs, MGs, ODCs, and A2B5^+^ cells. We validated a sexually dimorphic, male-specific activation of the NFκB signaling pathway, and RNA-seq signal of numerous genes is affected in a sexually dimorphic manner within NRs, MG, ODCs, and A2B5^+^ cells. NFκB and other inflammatory and immune related pathways identified in our study may be major mediators of fetal hypoxia-induced immature brain malformation and damage, which is associated with increased risk for neurological disorders in later life. The genes and pathways identified in this study are therefore potential therapeutic targets for attenuating fetal hypoxia-induced immature brain malformation and damage.

## Methods

### Experimental animals

Pregnant Sprague–Dawley (SD) rats (Charles River Laboratories, Portage, MI, USA) were randomly divided into two groups: (i) the normoxic control and (ii) hypoxic exposure (10.5% O_2_) from Day 15 to Day 21 of gestation, as we previously described [11, 34, 81–83]. The pups were sacrificed at neonatal day 7, and the brains were collected for cell sorting. All experimental procedures and protocols were approved by the Institutional Animal Care and Use Committee of Loma Linda University and followed the guidelines in the National Institutes of Health Guide for the Care and Use of Laboratory Animals.

### Cell sorting

Five types of brain cells (microglia, neurons, astrocytes, oligodendrocytes, A2B5^+^ glia precursor cells) were sorted from P7 pups using magnetic-activated cell sorting (MACS) technology. Briefly, brains were harvested from neonatal rats and then dissociated to single-cell suspensions using a Neural Tissue Dissociation Kit-T (Miltenyi Biotec) according to the manufacturer’s instructions. Myelin debris in single-cell suspensions was depleted by using Myelin Removal Beads II (Miltenyi Biotec) according to the manufacturer’s instructions. The unlabeled cell fraction after myelin removal was subjected to subsequent isolation of different neural cell populations, with the following MicroBead kits (Miltenyi Biotec): for microglia, CD11b/c MicroBeads; for astrocytes (precursor/immature cells), Anti-GLAST (ACSA-1) MicroBeads; and for oligodendrocytes (pre/immature/mature cells), Anti-O4 MicroBeads. For neurons (progenitor/immature cell), Anti-PSA-NCAM MicroBeads were used for sorting after depletion of A2B5^+^ glial progenitor cells using Anti-A2B5 MicroBeads.

### RNA isolation

Total RNA was isolated from each cell type using the Qiagen miRNeasy kit (Cat # 217004) following manufacturer’s protocol. In brief, QIAzol Lysis Reagent was used to disrupt and homogenize the cells and the lysate was incubated at room temp for 5 min. Then chloroform was added to the lysate, and shaken vigorously for 15 s. The mixture was re-incubated for 5 min and centrifuged for 15 min at 14000 RPM at 4 °C. The upper aqueous phase was collected to a fresh tube and 1.5 volumes of 100% ethanol was added. Then the sample was processed through a RNeasy Mini spin column. On-column DNase digestion was performed to effectively remove DNA during RNA purification by using the RNase-Free DNase set (Qiagen, cat# 79254) according to the manufacturers’ instructions. After final column wash, total RNA was eluted out of mini column membranes using 30 µl nuclease free water (Qiagen). Integrity of RNA was confirmed by determining RIN number. Total RNA was delivered to the Center for Genomics at Loma Linda University for library construction and RNA sequencing.

### RNA-Seq library construction

The Ovation® Rat RNA-Seq System (NuGEN Technologies, San Carlos, CA) was used per manufacturer’s instructions to construct RNA-seq libraries. 100 ng of total RNA was used as input. First and second strands of cDNA were synthesized from total RNA (100 ng) spiked with 1 µl of 1:500 diluted ERCC ExFold RNA Spike-In Mix 2 (Life Technologies, Carlsbad, CA) at a final concentration of 1%. Following primer annealing and cDNA synthesis, end-repair, adaptor index ligation, and strand selection was conducted. Barcodes with unique indices was used per sample for multiplexing. Ribosomal RNA depletion was performed by using custom InDA-C primer mixture SS5 V8 for rat. Finally, libraries were amplified for 13 cycles (Mastercycler® pro, Eppendorf, Hamburg, Germany), and purified with Agencourt XP beads (Beckman Coulter, Indianapolis, IN).

### Library quantification, quality control (QC) and sequencing

The final amplified libraries were purified using Agencourt XP beads and quantified using the Qubit dsDNA HS Kit on Qubit 3.0 Fluorometer (Life Technologies, Carlsbad, CA). Quality and peak size were determined with the D1000 ScreenTape on Agilent 2200 TapeStation (Agilent Technologies, Santa Clara, CA). Final libraries were pooled and sequnced on an Illumina NextSeq 550 or HiSeq 4000 (Illumina, Inc., San Diego, CA). Paired-end reads of 75 bp were generated.

### RNA-seq data analysis

Raw fastq files were trimmed to remove adaptors and low quality bases using trim_galore [84] version 0.6.6., a wrapper for cutadapt [85] (version 2.8). Trimmed fastq files were then mapped to the rn7 reference genome using tophat (version 2.1.1) [86] using the refFlat gene annotation for rn7, obtained from the UCSC table browser [87]. A custom script was used to select only reads that mapped uniquely to one location on the genome for downstream analysis by taking only reads marked with “NH:i:1” in the tophat2 output.bam files. Normalized gene expression (FPKM) was quantified for each sample using cufflinks version 2.2.1 [88]. Differential gene expression analyses were performed using cuffdiff version 2.2.1 [89] using q < 0.05 and |log_2_FC|> 1 as the cutoffs for differential expression. To determine genes with interaction effects between sex and hypoxia, a two-way analysis of variance (ANOVA) was performed on FPKM values of genes output by cufflinks, using sex and hypoxia as the factors. The analysis was done on each cell type independently in R version 4.0.3, using the function “aov()”, with the input argument “scale.unit = F”. Interaction P-values from the ANOVA were input into the R function “qvalue()”, and genes with q < 0.05 for the interaction term were used in the final list of interaction genes.

### Statistical comparison of the number of male vs. female hDEGs

For each cell type, a 2X2 contingency matrix,

$$\begin{array}{ll} {\text{X}}_{11} & {\text{X}}_{12} \\ {\text{X}}_{21} & {\text{X}}_{22} ,\end{array}$$

was constructed, where X_11_ = # of female hDEGs, X_12_ = # of non-hDEGs in female, X_21_ = # of male hDEGs, X_22_ = # of non-hDEGs in male. Non-hDEGs are the total number of genes analylzed minus the number of hDEGs in the given sex and cell type. Fisher’s Exact Test was then calculated on each 2X2 contingency matrix in order to obtain a P-value for the null hypothesis that the proportion of genes that were hDEGs in male is the same as the proportion in female.

### Principal component analyses

FPKM values output by cufflinks, as described above, were used as inputs for the PCA. The analysis was done in R version 4.0.3. The R function “aov()” was used to perform a linear analysis of variance (ANOVA) at each gene, using each sample as a factor. The R function “qvalue()” was then used to calculate ANOVA q-values for all genes, based on the P-values output by “aov()”. For genes with q < 0.05 in the ANOVA, the FPKM of all replicates were then input into the R function “PCA()”, with the input argument “scale.unit = F”, in order to generate PCA plots.

### Pathway analyses

DEG symbols for each cell type were input separately into the Enrichr pathway analysis online tool [90]. Enriched pathways in this paper are from the KEGG 2019 Mouse pathway analysis in the “legacy” tab of the Enrichr website. Pathway shown in this page are rat-specific diagrams (species code rno) from the Kyoto Encyclopedia of Genes and Genomes website [91].

### Transcription factor motif enrichment analyses

Annotated enhancers for rat ODCs, ODC precursors, and NRs were obtained from EnhancerAtlas2.0 [92]. EnhancerAtlas2.0 does not contain rat MG enhancers, so instead we used published DNAse-I Hypersensitive Sites (DHSSs) taken from rat MG under a variety of conditions associated with inflammation [93]. Both the DHSSs and enhancers from EnhancerAtlas2.0 were in rn5 coordinates, and so all were converted to rn7 coordinates using UCSC liftOver [94].

For each brain cell type, we compiled a list of expressed genes by taking the set of genes with FPKM > 1 in at least one treatment for the cell type (hypoxia or control), with replicates of each sample pooled before calculating FPKM values in cufflinks as described above. All regions within 500 base pairs from any transcription start site (TSS) of an expressed gene were removed from subsequent analysis, as it would be ambiguous in those cases whether the sites are acting as enhancers or promoters. We also removed enhancers/DHSSs that were > 10 kilobases from the nearest TSS of an expressed gene.

We extracted the DNA sequences at the remaining enhancers/DHSSs, using bedtools getfasta (version 2.27.1. TF motif scanning was then done at these sequences using fimo [95] version 5.3.0, using a combination of the jolma2013.meme, JASPAR_CORE_2014_vertebrates.meme, and uniprobe_mouse.meme TF motif data files provided by fimo for motif scanning. Then, in each cell type, for each TF that was expressed in the cell type, we used Fisher’s exact test to calculate the P-value for the enrichment of the number of enhancers containing a motif for the TF at enhancers near DEGs relative to enhancers/DHSSs near all expressed genes (with proximity defined as within 10 kilobases of the TSS).

### qRT-PCR assay

Total RNA was converted to cDNA using Maxima First Strand cDNA Synthesis kit with dsDNase (Thermo Fisher) following manufacture’s protocol. Briefly, 1 µg total RNA in 10 µl containing 1X DNAse buffer and 1 µl DNase was incubated for 2 min at 37 °C, followed by incubation on ice for 5 min. 10 µl of 1X Maxima Enzyme Mix was next added to it and incubated for 10 min at 25 °C followed by 30 min at 50 °C, 85 °C for 5 min and then hold at 4 °C until ready for QPCR. The specific qPCR primers for target genes were designed using Primer Blast tool. https://www.ncbi.nlm.nih.gov/tools/primer-blast/index.cgi?LINK_LOC=BlastHome. All primers were synthesized at the Integrated DNA Technologies. The cDNA template (10 ng), forward and reverse primers each at 0.5 µM, IQ SYBR Green Supermix (BIO-RAD) were mixed in a total of 20 µl volume. β-Actin primers were used as an internal control. Each qPCR was done for 30 cycles. The relative expression of gene of interest was calculated by the 2^−ΔΔCT^ method [96] and is presented as the fold modulation in hypoxia relative to the control. PCR primer details are in the supplemental materials (Additional file 2: Table S4).

### Nuclear extraction

The Nuclear Extract Kit (Active Motif, Cat # 40010) was used to isolated nuclear from the brain of rat pups according to manufacturer’s instruction. The brain hemisphere was transferred to a pre-chilled glass Dounce homogenizer with 1 ml of Hypotonic Buffer supplemented with protease inhibitors, phosphatase inhibitors, DTT, and detergent. The tissue was homogenized 30 times on ice bath until dispersed to a homogeneous cell suspension. Then the cell suspension was incubated 15 min on ice, vortexed and re-incubated on ice for 15 min. The cell suspension was first centrifuged at 2500 RPM for 10 min to get rid of tissue debris. The collected supernatant was centrifuged at 14000 RPM for 30 min again. The pellet was resuspended in 100 µl Complete Lysis buffer containing DTT, AM1, protease inhibitor, detergent and vortexed at maximum strength for 20 s followed by incubation on ice. This procedure was repeated 10 times. Finally, the content was centrifuged for 20 min at 14000RPM at 4 °C. The supernatant was the Nuclear Extract (NE), which was stored at −80 °C. The protein concentration of each NE was determined by Bradford Method before TransAM NFκBp65 assay.

### TransAM NFκBp65 assay

The TransAM NFκBp65 kit (Active Motif, Cat # 40096) was used to measure the activation of NFκB pathway according to the manufacture’s instruction. Complete Lysis Buffer (CLB) and Complete Binding Buffer (CBB) were prepared using the Kit reagents. CBB (30 µl) was first added to each well followed by NE (20 µg protein in 20 µl CLB) prepared from each tissue. The assay was done in triplicate. For negative controls, only 20 µl CLB was added to 30 µl of CBB. For positive control wells, 2.5 µg of stimulated Jurkat cell nuclear extract (kit component) was used. When all samples were added to the wells, the adhesive cover was added to seal the plate. The plate was incubated at room temperature for 1 h with gentle rocking at around 100 RPM. Then, each well was washed 3 times with 200 µl wash buffer followed by 100 µl of NFκB antibody (1:1000 dilution in antibody binding buffer). The plate was sealed and incubated at room temperature for 1 h with no agitation. After wash, 100 µl of HRP conjugated secondary antibody (1:1000 dilution in antibody binding buffer) was added and incubated at room temperature for another 1 h with no agitation. After wash, 100 µl of Developing solution was added to each well, incubated for 5 min at room temp protected from light, until the blue color develops, and the colorimetric reaction was stopped by adding stop solution. The absorbance was read in a 96 well plate reader at 450 nm with a reference wave-length of 655 nm.

### Statistical analysis

For non-bioinformatic data analysis, data were presented as mean ± SEM, and were analyzed using Student’s *t*-test or two-way ANOVA followed by Tukey’s post hoc test. *P*-value < 0.05 was considered statistically significant. Experimental number (*n*) represents brains of animals from different dams.

## Supplementary Information

**Additional file 1: Figure S1.** Quality control of RNA-seq data. **Figure S2.** Related to Fig. 2. **Figure S3.** Related to Fig. 3. **Figure S4.** Related to Fig. 4.

**Additional file 2: Table S1.** Hypoxia vs. Normoxia DEG (hDEG) counts. **Table S2.** Male vs. Female DEG counts**. Table S3.** Interaction DEG counts. **Table S4.** PCR primer details.

## Data Availability

RNA-seq data that support the findings of this study have been deposited in NCBI GEO with the accession code GSE216946. All custom scripts used to process and analyze the data and to obtain the results described in this paper have been deposited in the GitHub repository (https://github.com/ikremsky/Scripts-for-Fetal-hypoxia-results-in-sex--and-cell-type-specific-alterations-in-neonatal-transcript).
