# Supplementary material for: Fetal hypoxia results in sex- and cell type-specific alterations in neonatal transcription in rat oligodendrocyte precursor cells, microglia, neurons, and oligodendrocytes
Source: Cell Biosci. 2023 Mar 17;13:58. doi: 10.1186/s13578-023-01012-8 (PMC10022003; doi:10.1186/s13578-023-01012-8)
Supplement: Supplementary file 1 — Additional file 1: Figure S1. Quality control of RNA-seq data. Figure S2. Related to Fig. 2. Figure S3. Related to Fig. 3. Figure S4. Related to Fig. 4. [file 13578_2023_1012_MOESM1_ESM.docx]

**Figure S1**

**
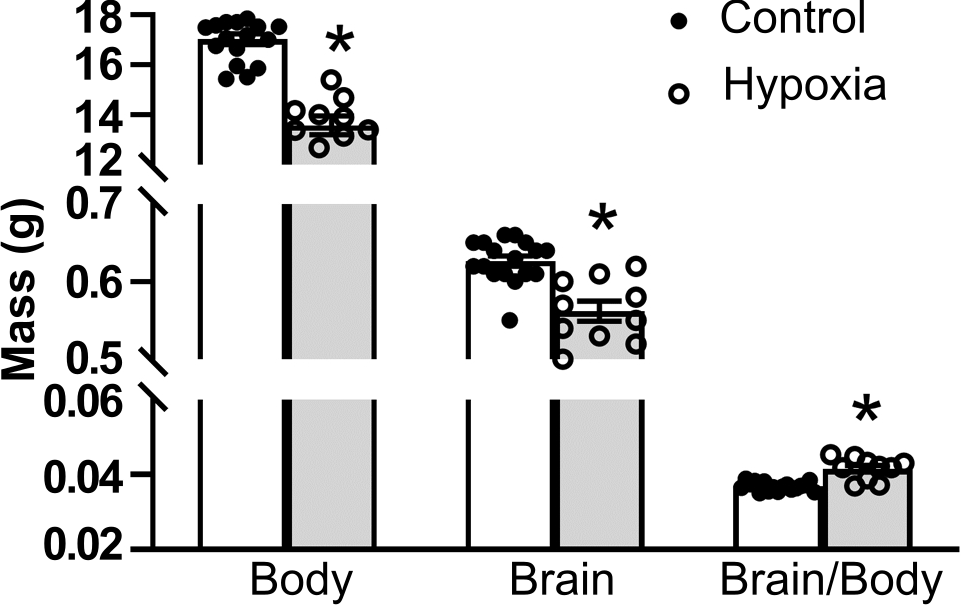
**

**
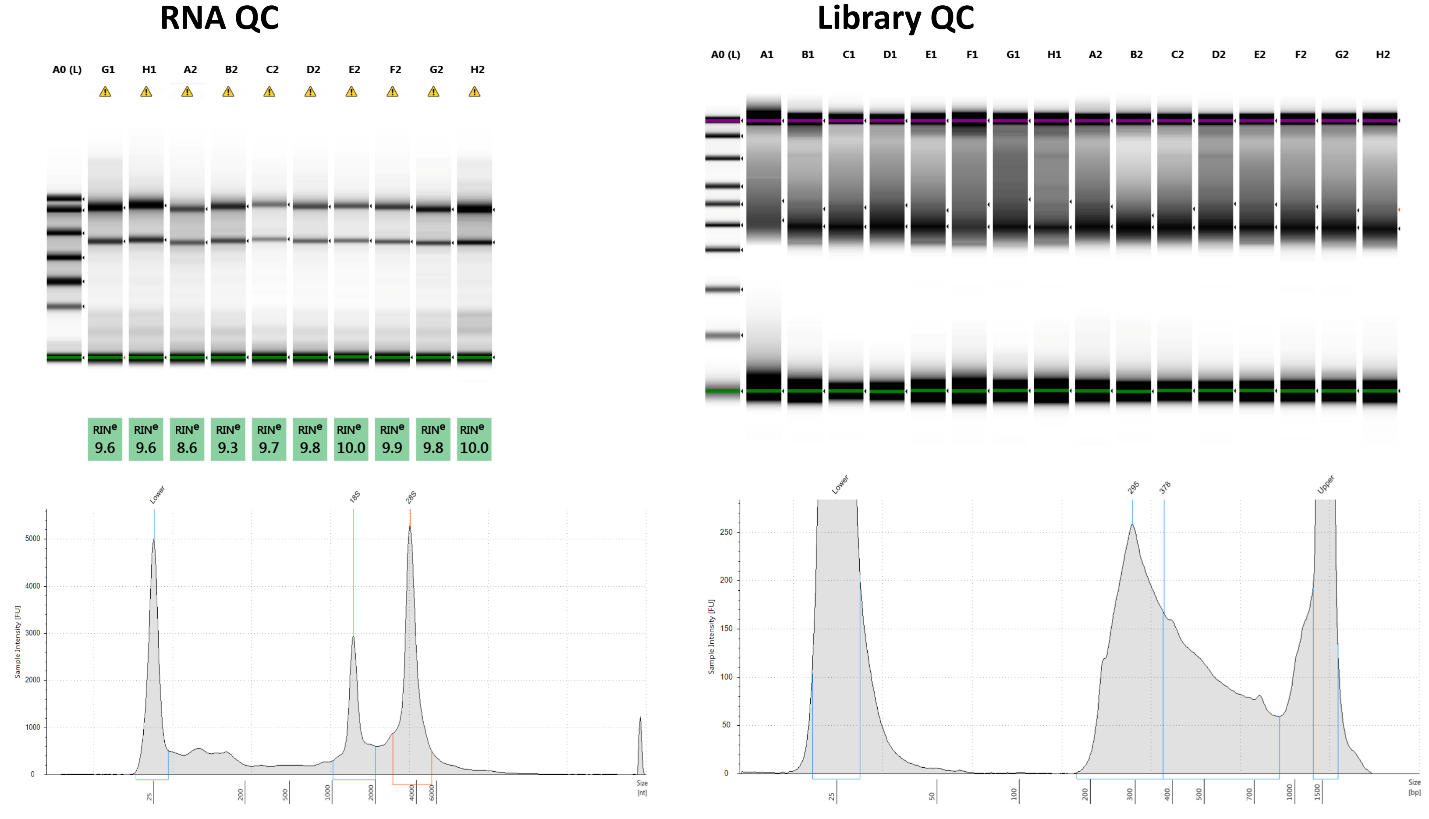
B.**

**Figure S1 (Cont.)**

**
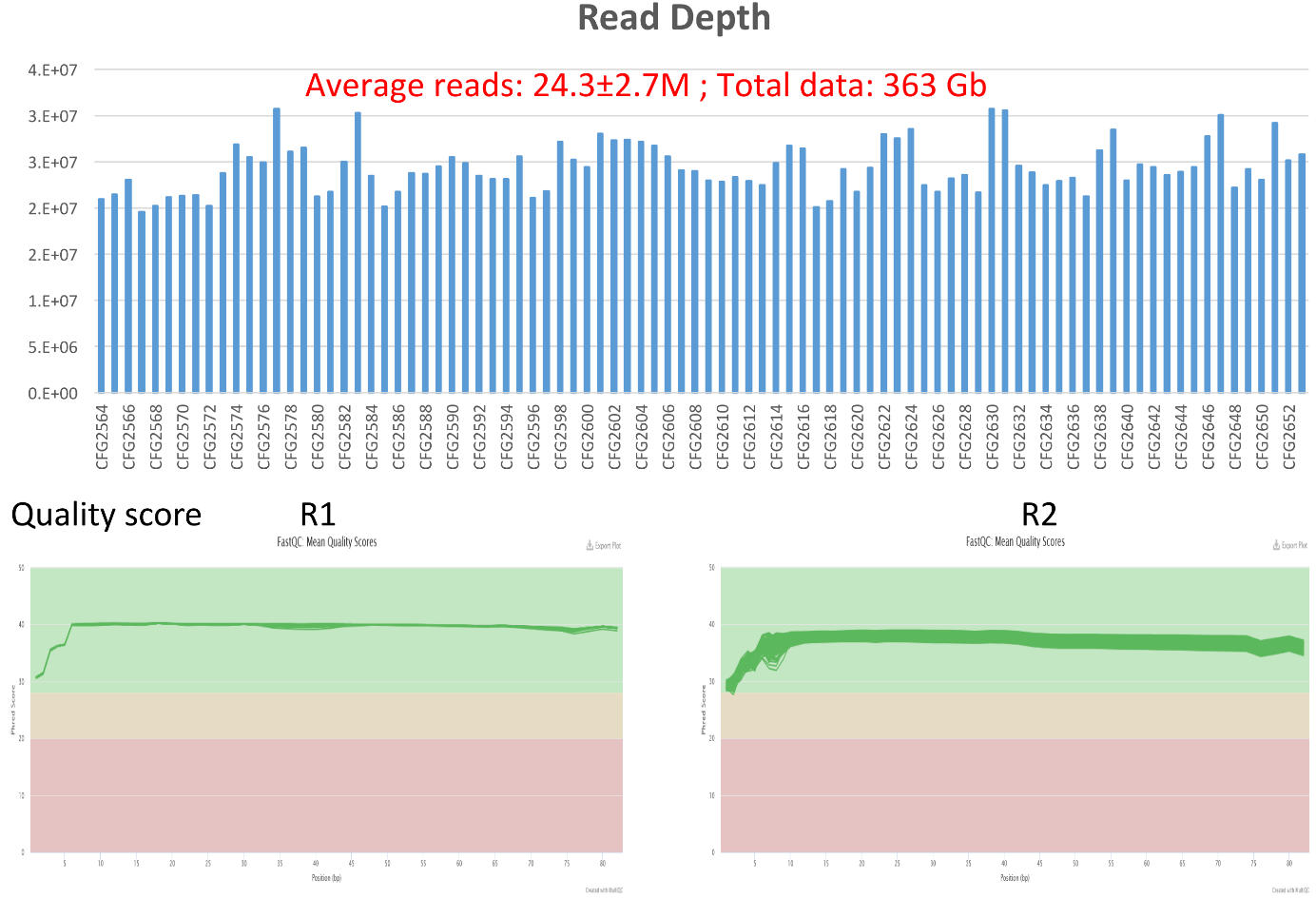
C.**

**Figure S1 (Cont.)**

**Pearson Correlation Coefficient**

**
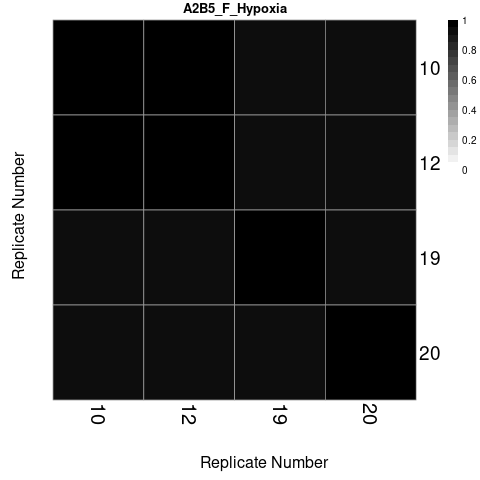

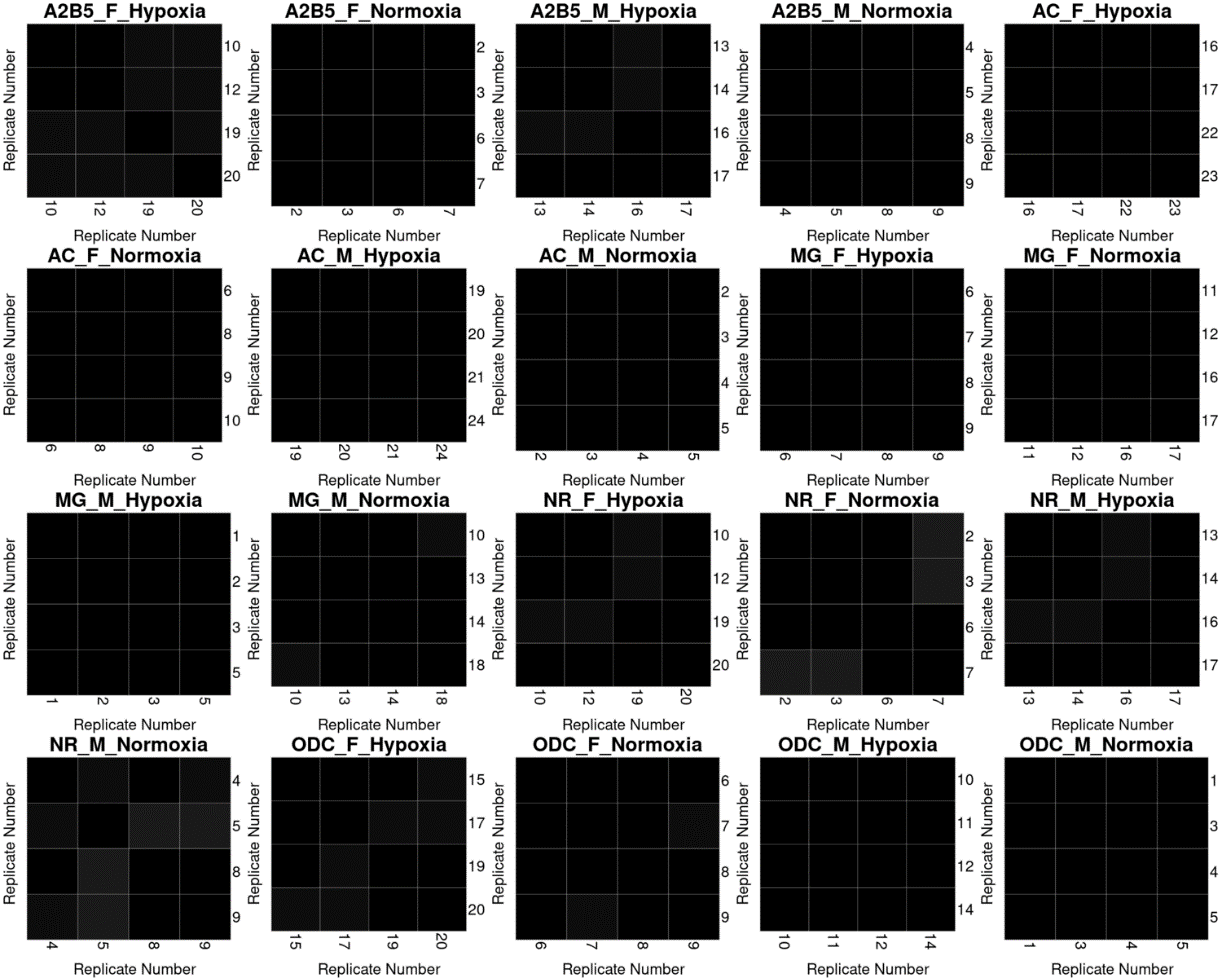
D.**

**
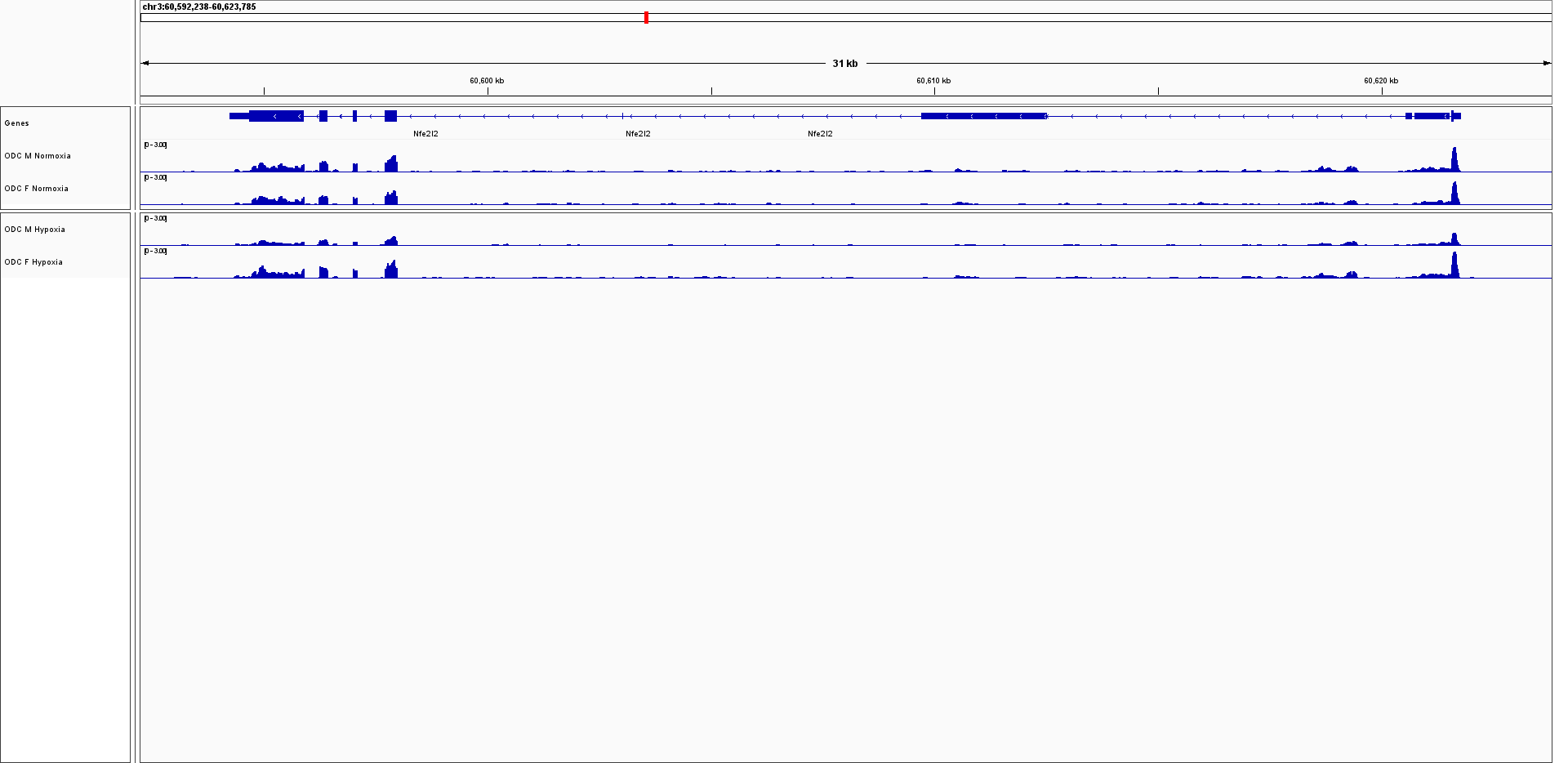
E. ODC_NFE2I2**

**Figure S1 (Cont.)**

**
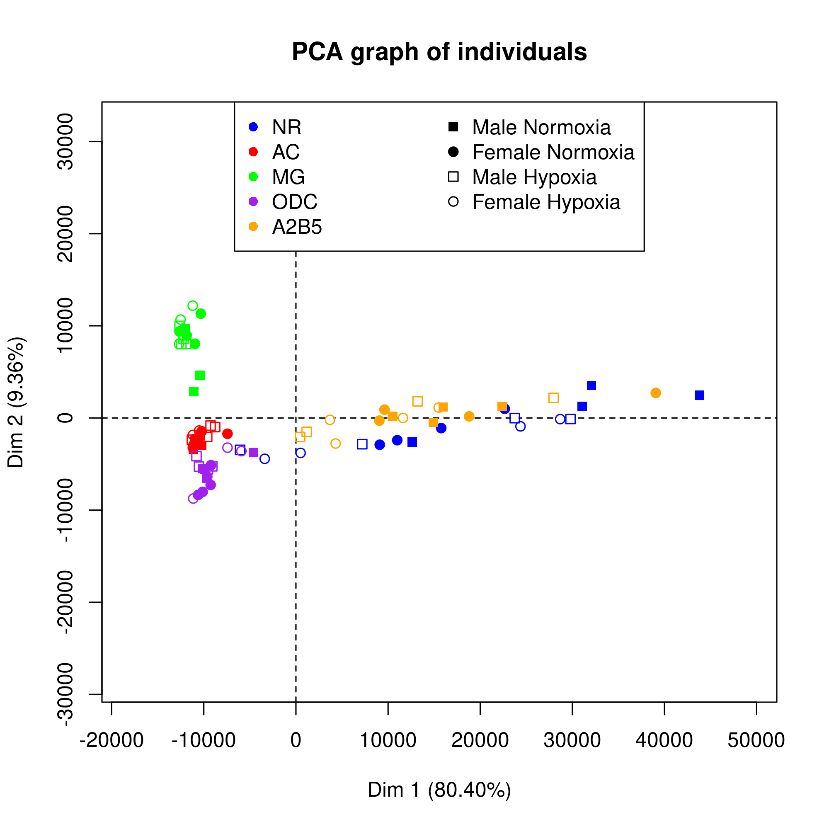
F.**

**Figure S1.**

Quality control of RNA-seq data. **A** Plots showing the average (tops of bars), standard error (error bars), and distribution (individual points) of body mass, brain mass, and the ratio of brain/body masses. Masses are shown in grams. P-values are determined by t-test. *P < 0.05, hypoxia versus control. **B** RNA quality control and library quality control. **C** Total read depth per sample (top), and per-base quality scores of all samples (bottom). **D** Heatmaps showing Pearson’s correlation coefficient between the indicted replicates of the indicated samples, based on log_10_(FPKM) values. **E** Genome browser view showing RPM-normalized gene expression values of Nfe212, with replicates of each sample pooled. **F** The first two principal components in the PCA of all samples combined.

**Figure S2**

**
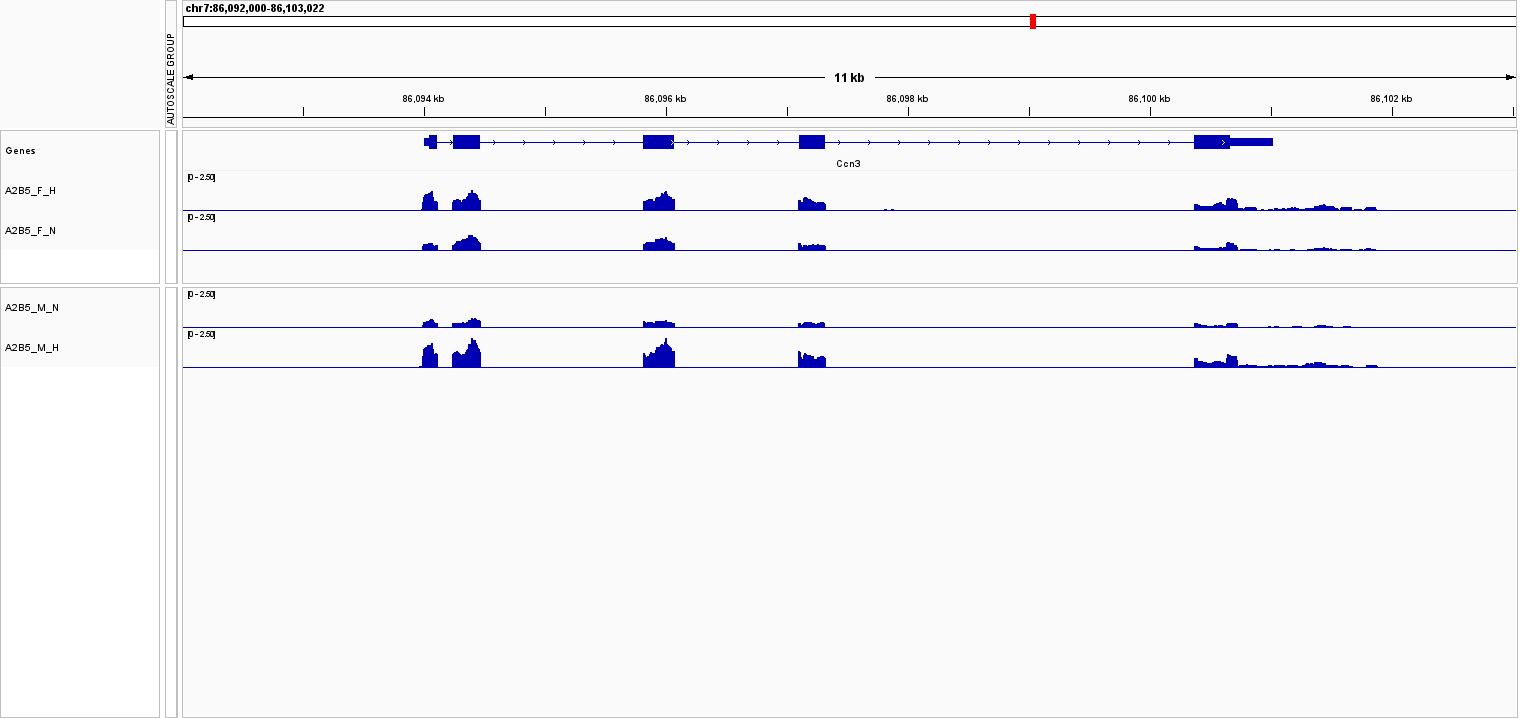
A. A2B5**

**
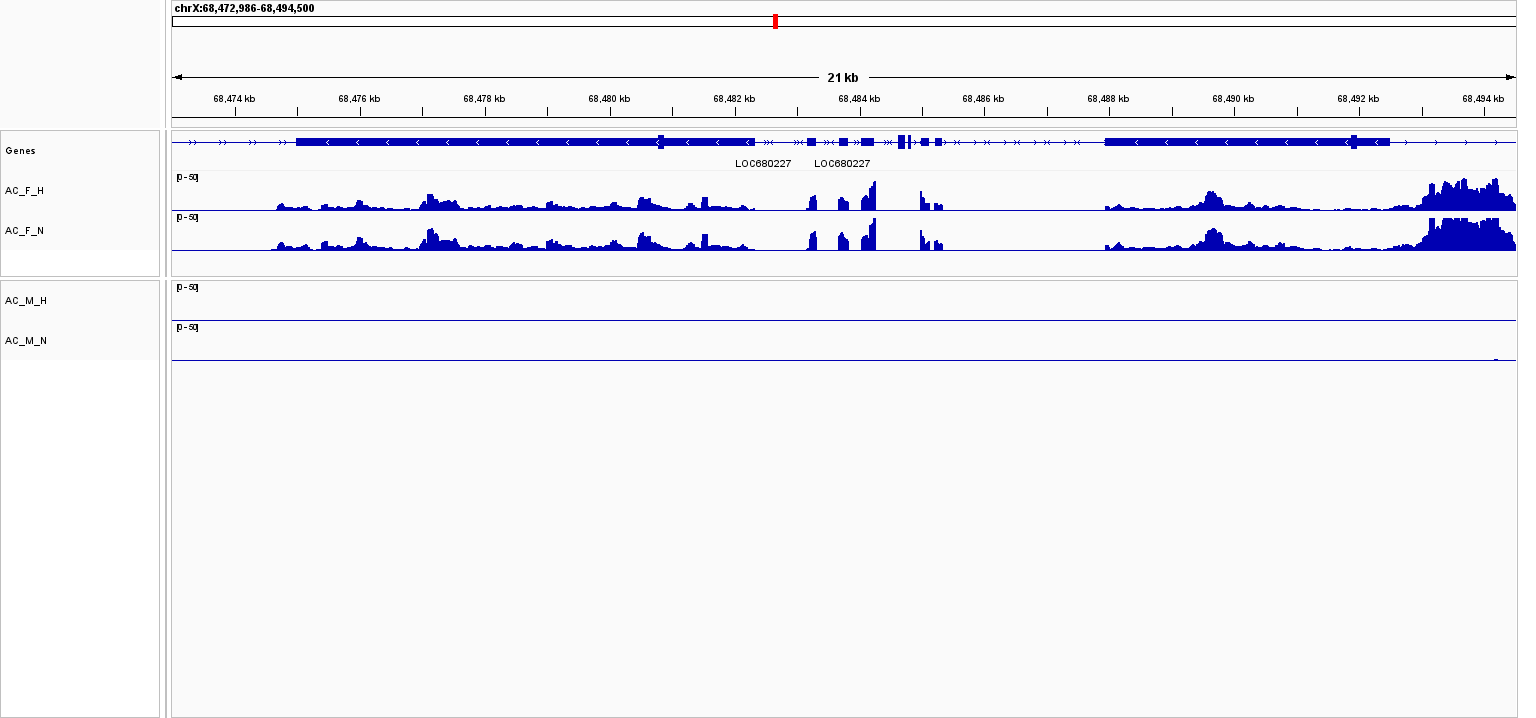
B. AC**

**
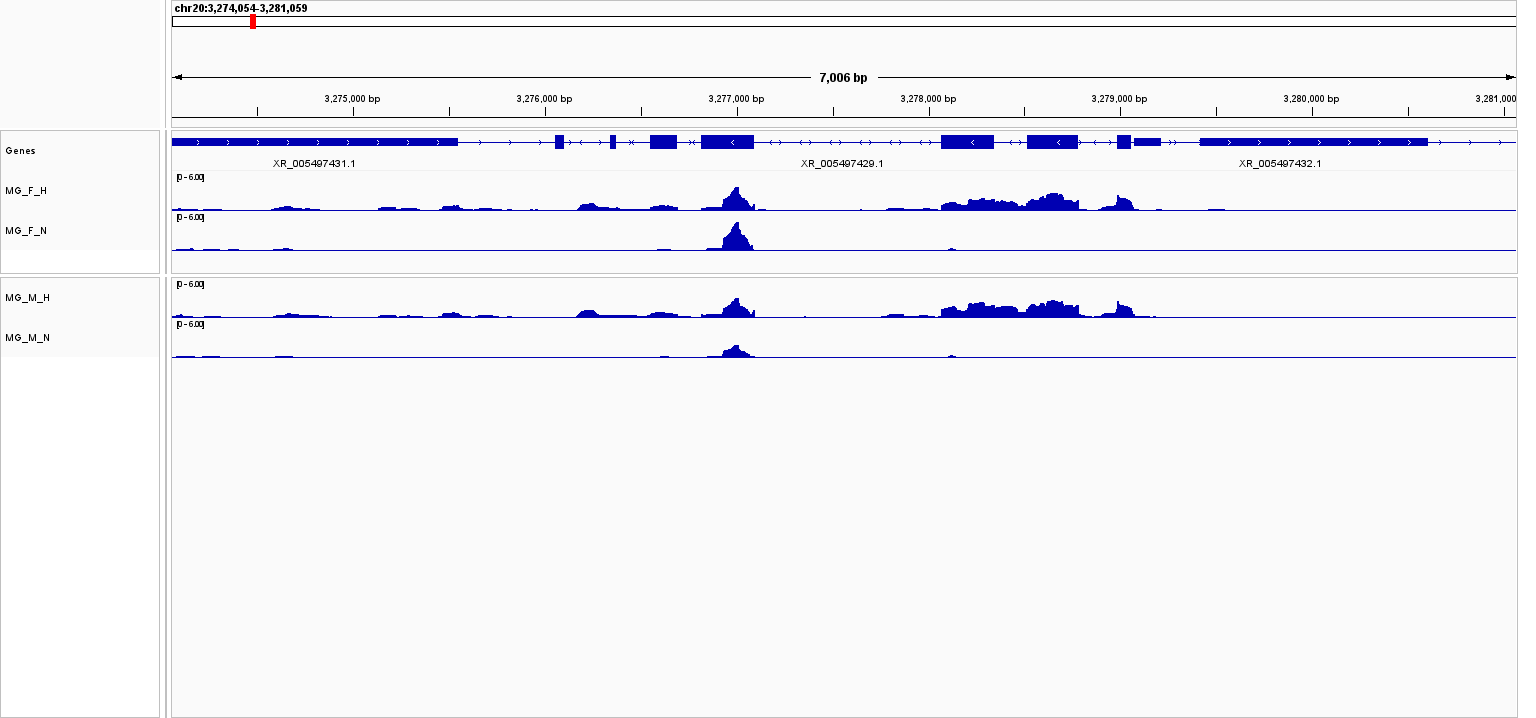
C. MG**

**Figure S2 (Cont.)**

**
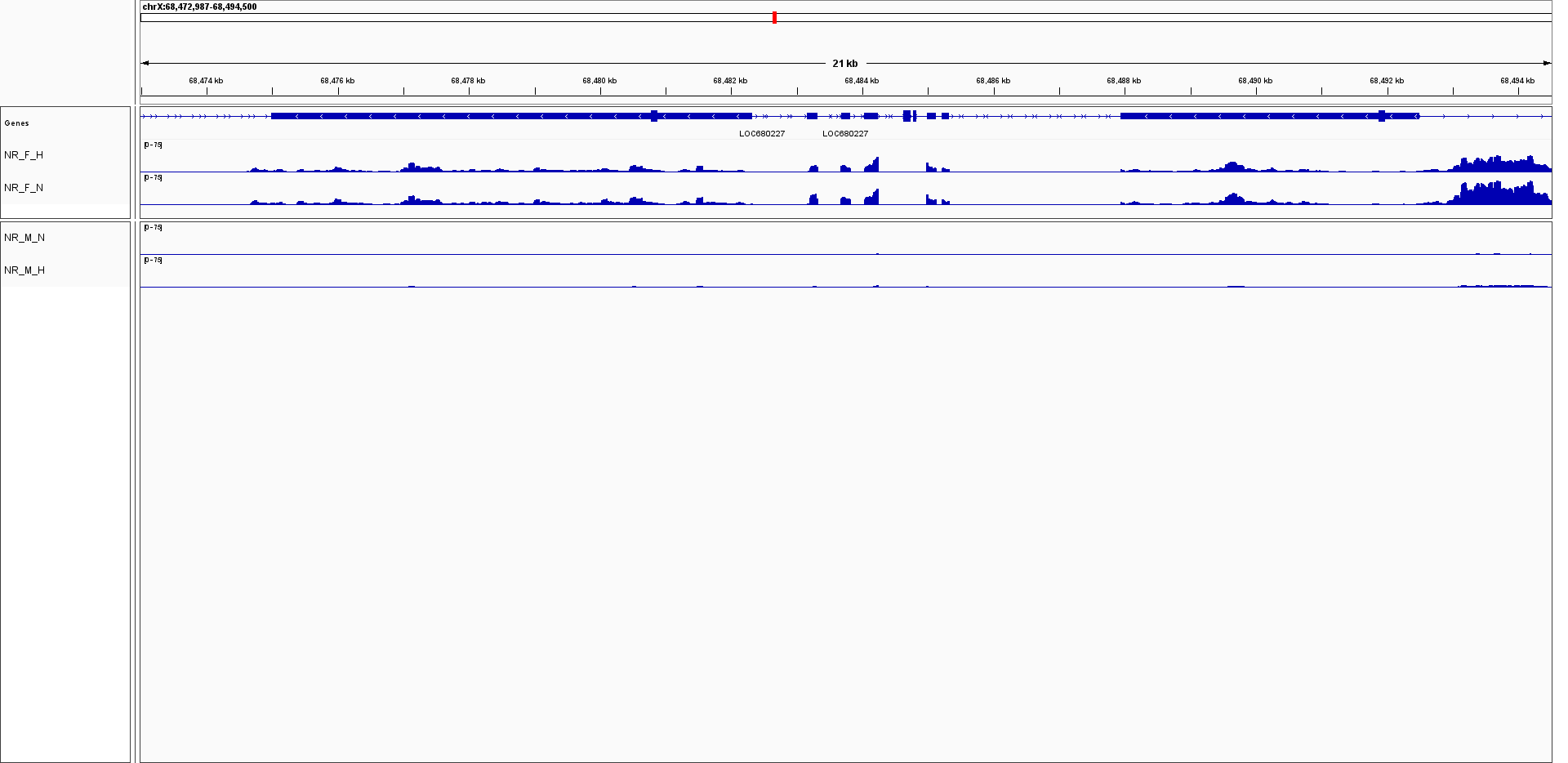
D. NR**

**
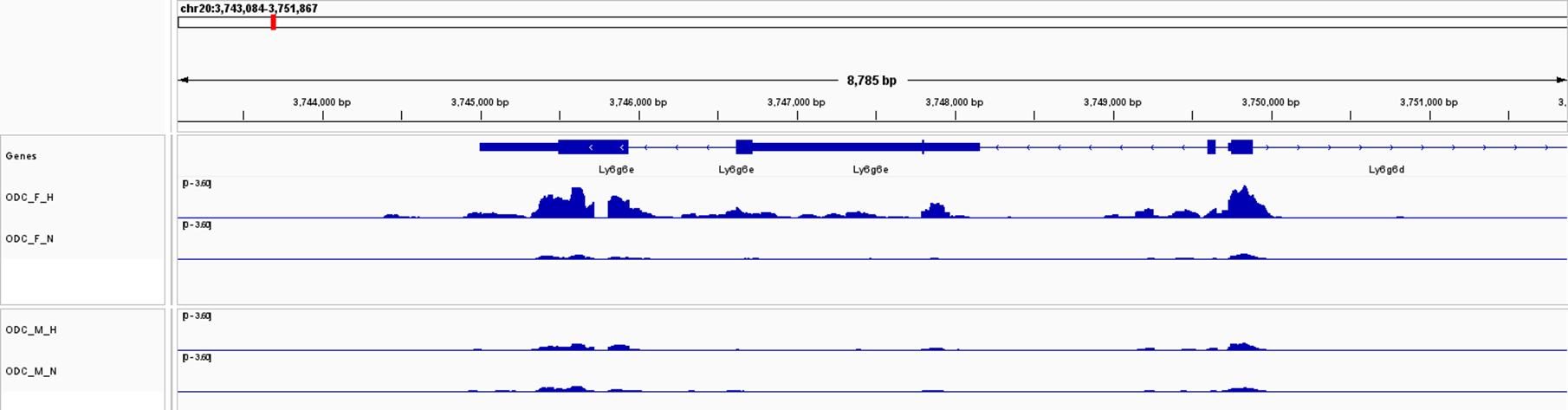
E. ODC**

**Figure S2.**

Related to Figure 2. Genome browser images showing RPM-normalized gene expression values of samples with replicates pooled. These are genes that illustrate the pattern of variation indicated in the PCA plots of Figure2 for **A** A2B5^+^ cells **B** ACs **C** MG **D** NRs **E** ODCs. M=Male; F=Female; N=Normoxia; H=Hypoxia.

**Figure S3**

1. MG-M


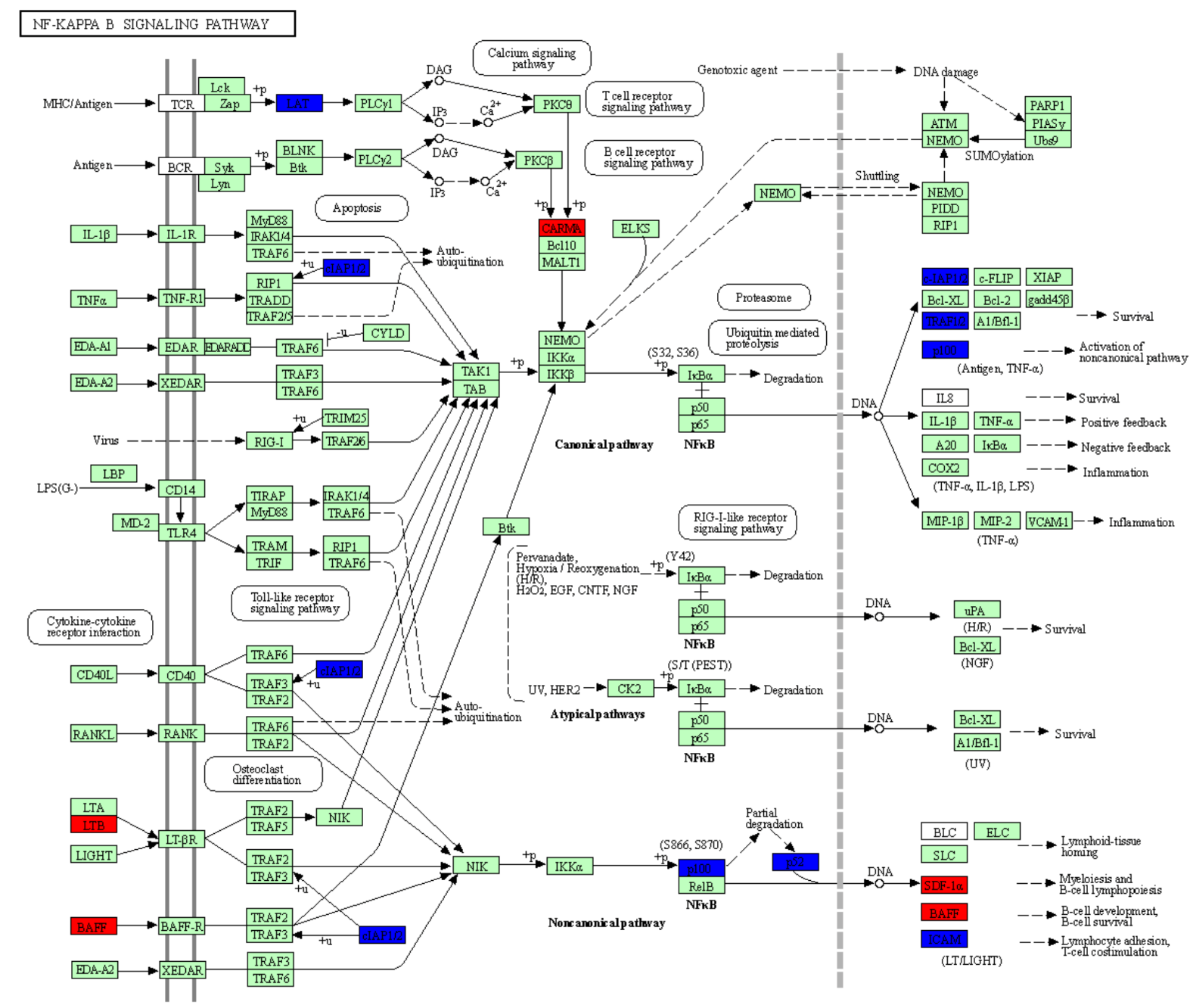
**Figure S3 (Cont.)**

**B.** MG-M

**
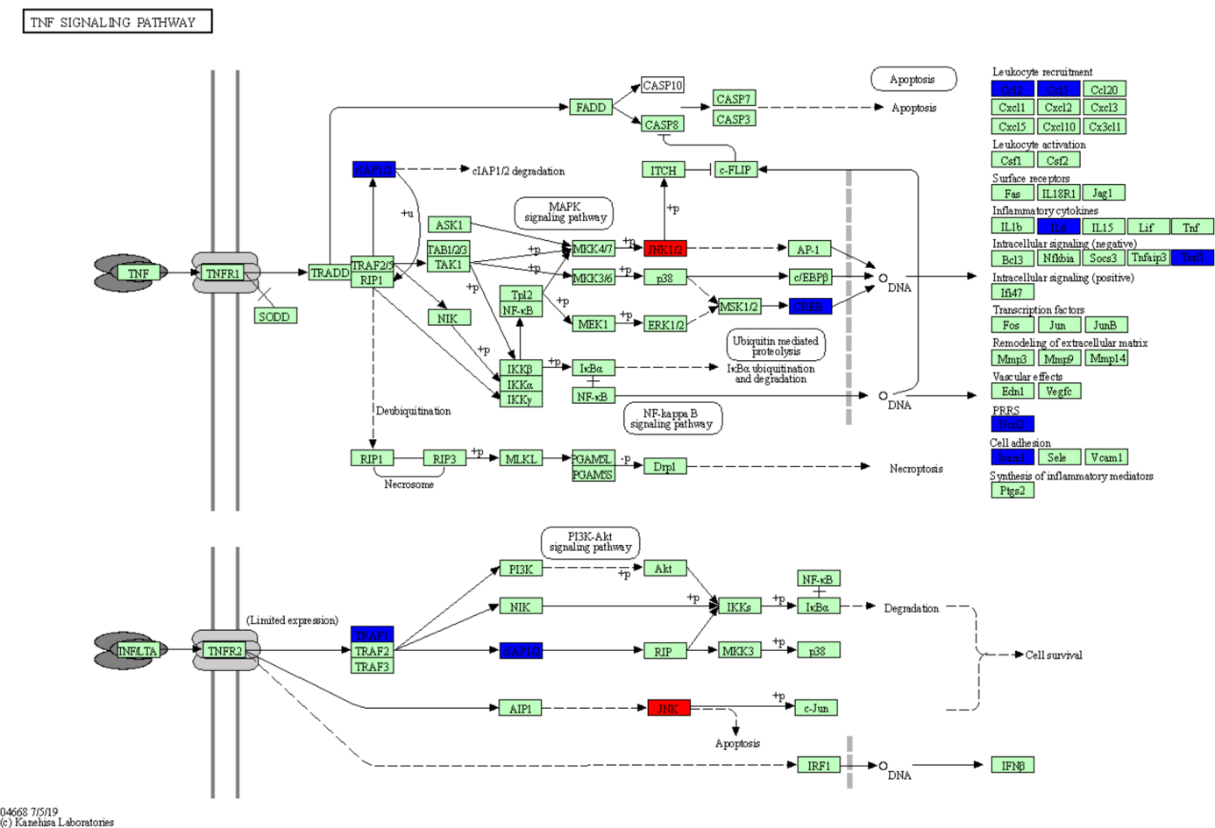
**

**Figure S3 (Cont.)**

**
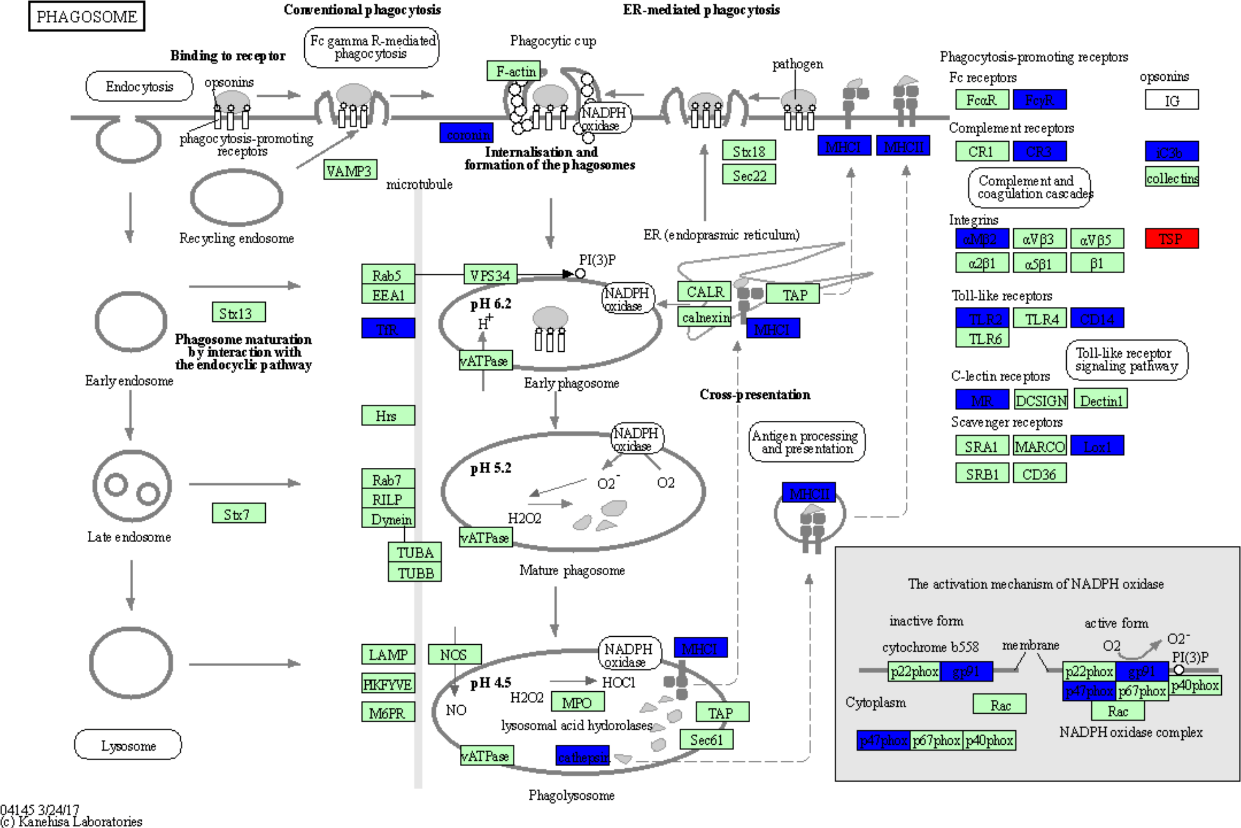
C.** NR-F

**
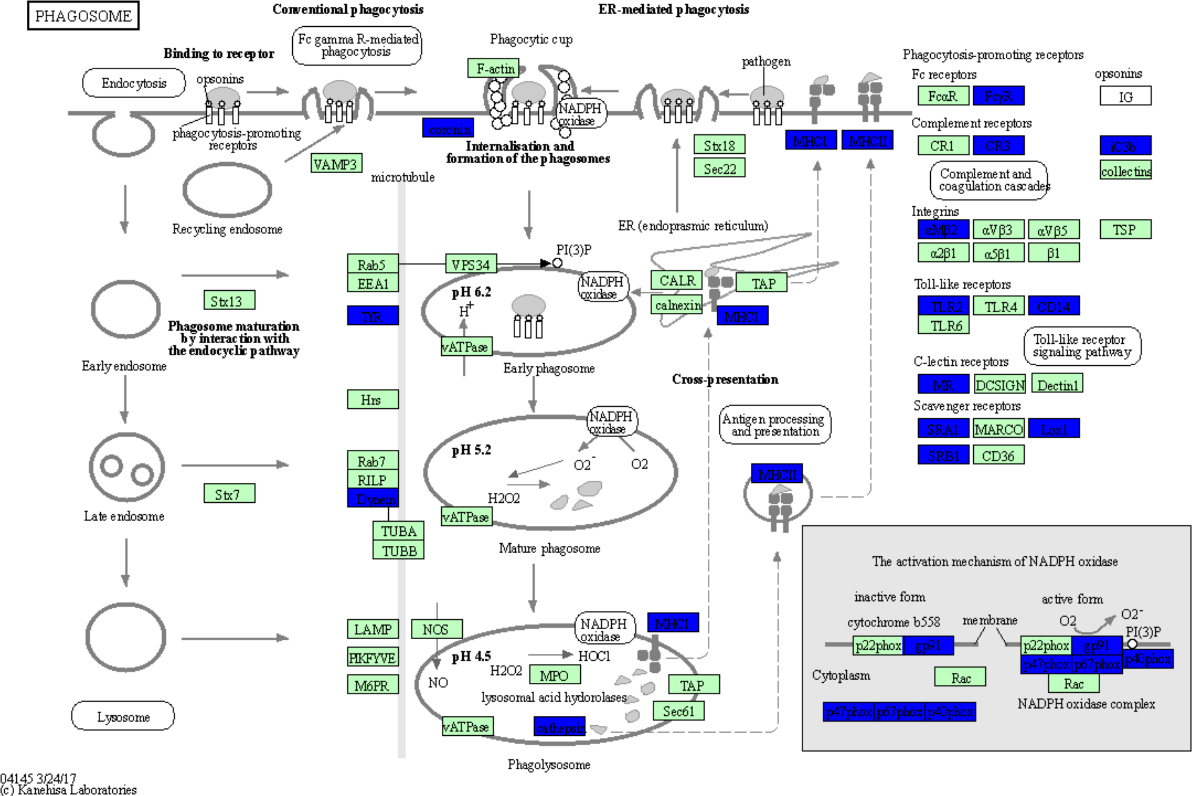
D.** NR-M

**Figure S3 (Cont.)**

**
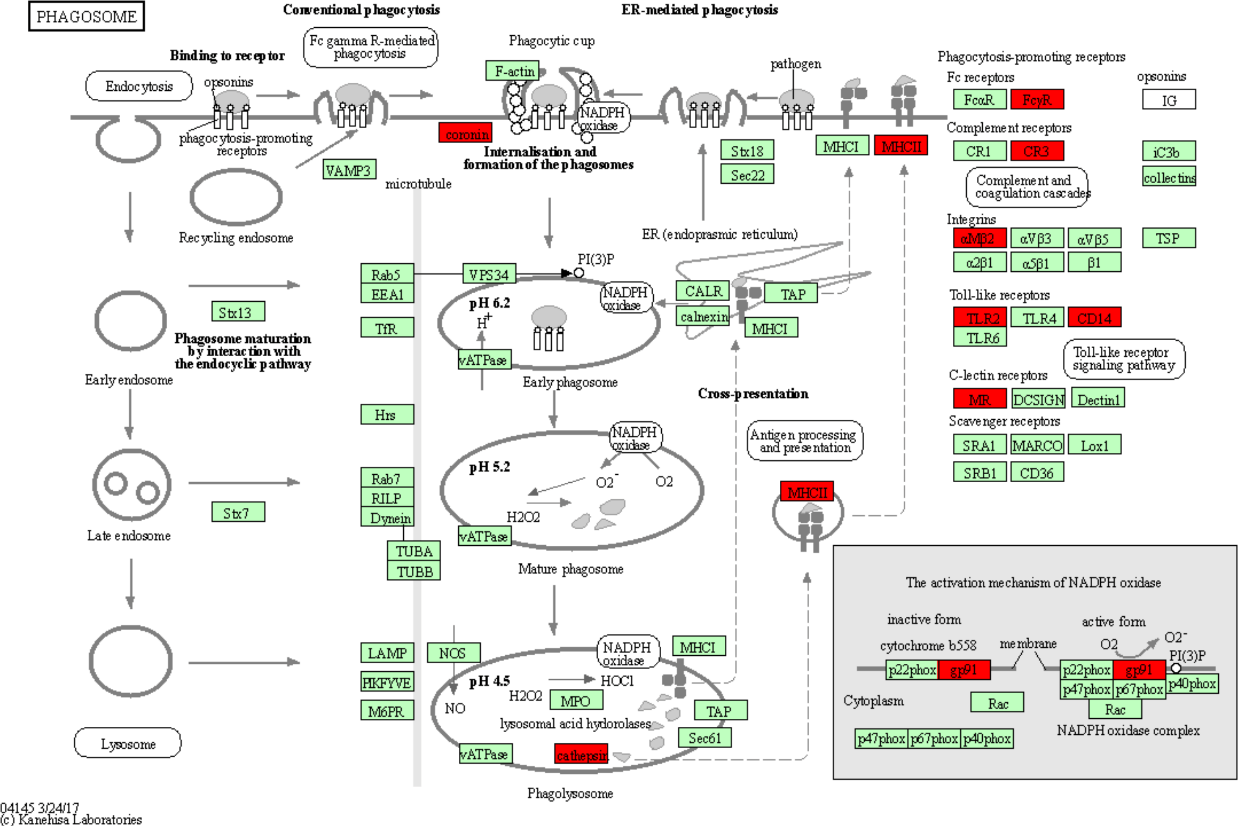
E.** ODC-F

**
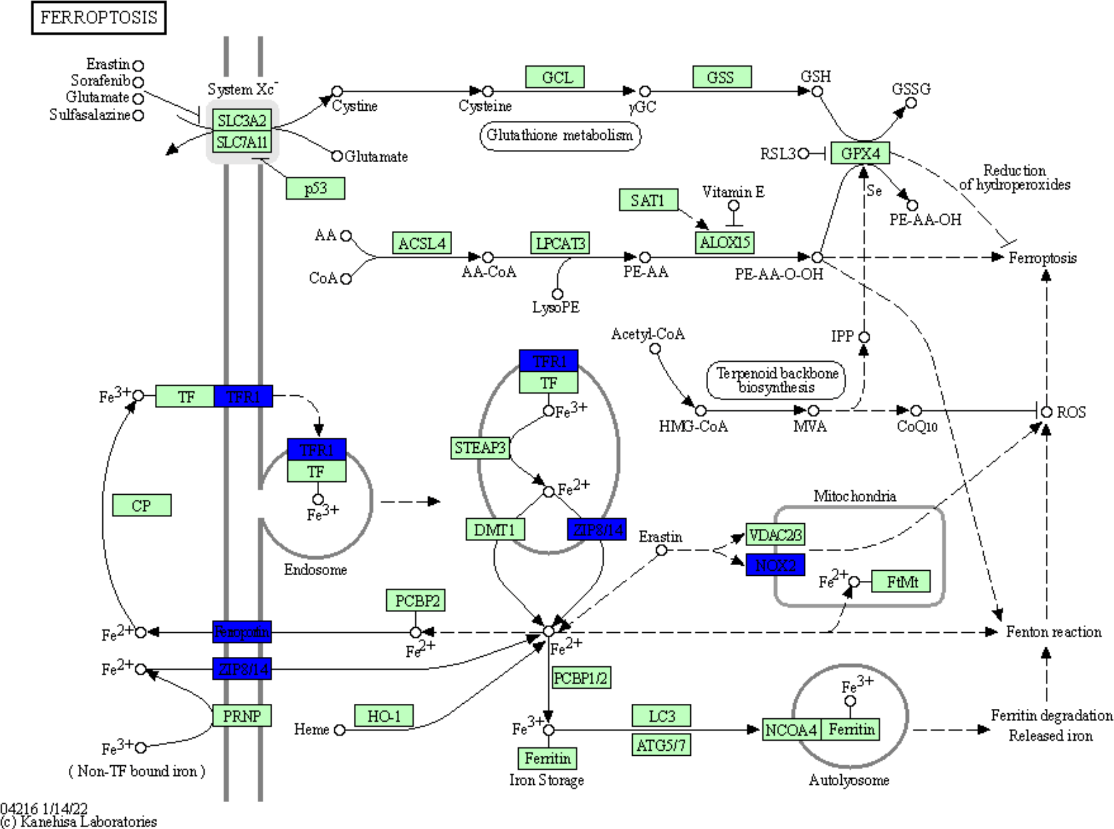
F.** NR-F

**Figure S3 (Cont.)**

**G.** NR-M

**
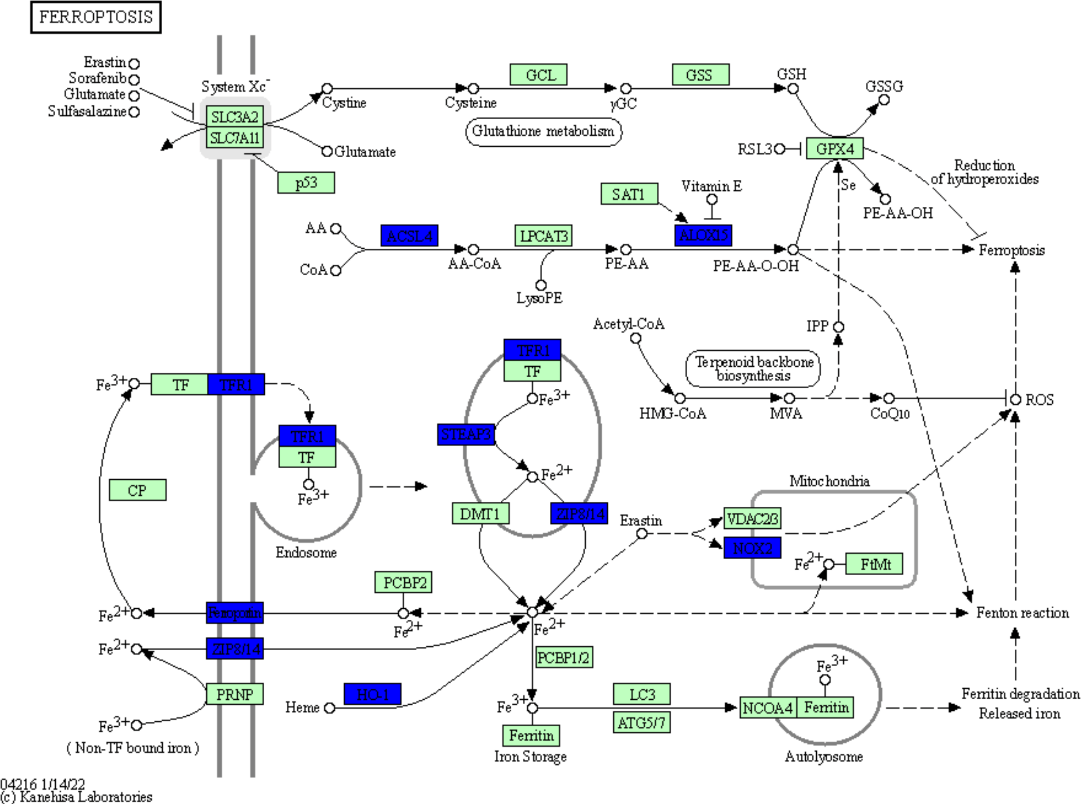
**

**
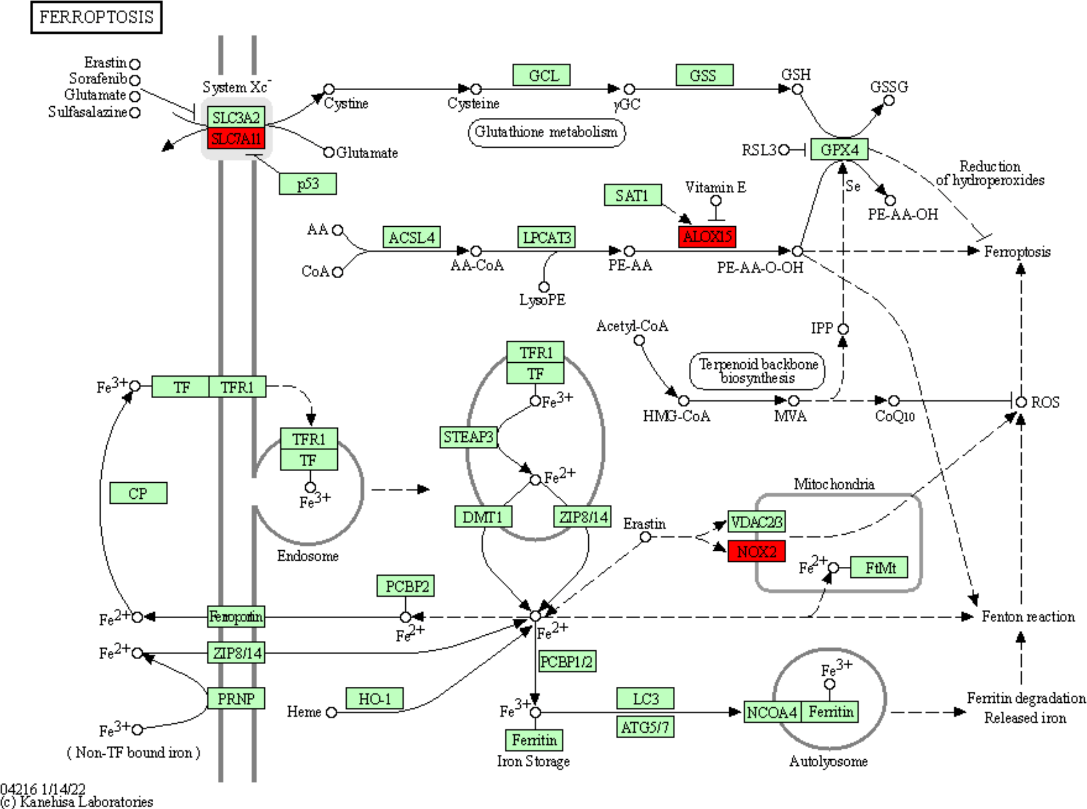
H.** ODC-F

**Figure S3 (Cont.)**

**I.** NR-F

**
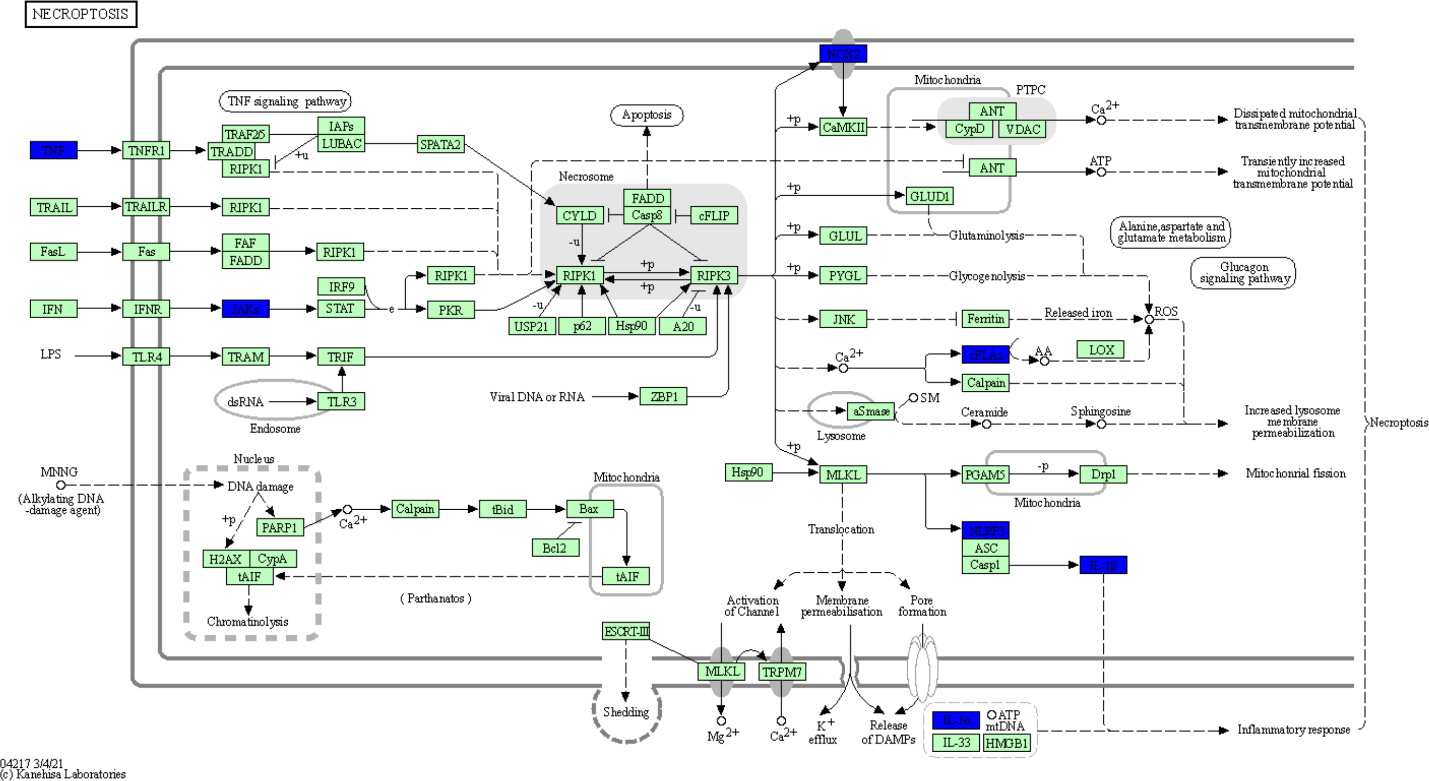
**

**J.** NR-M

**
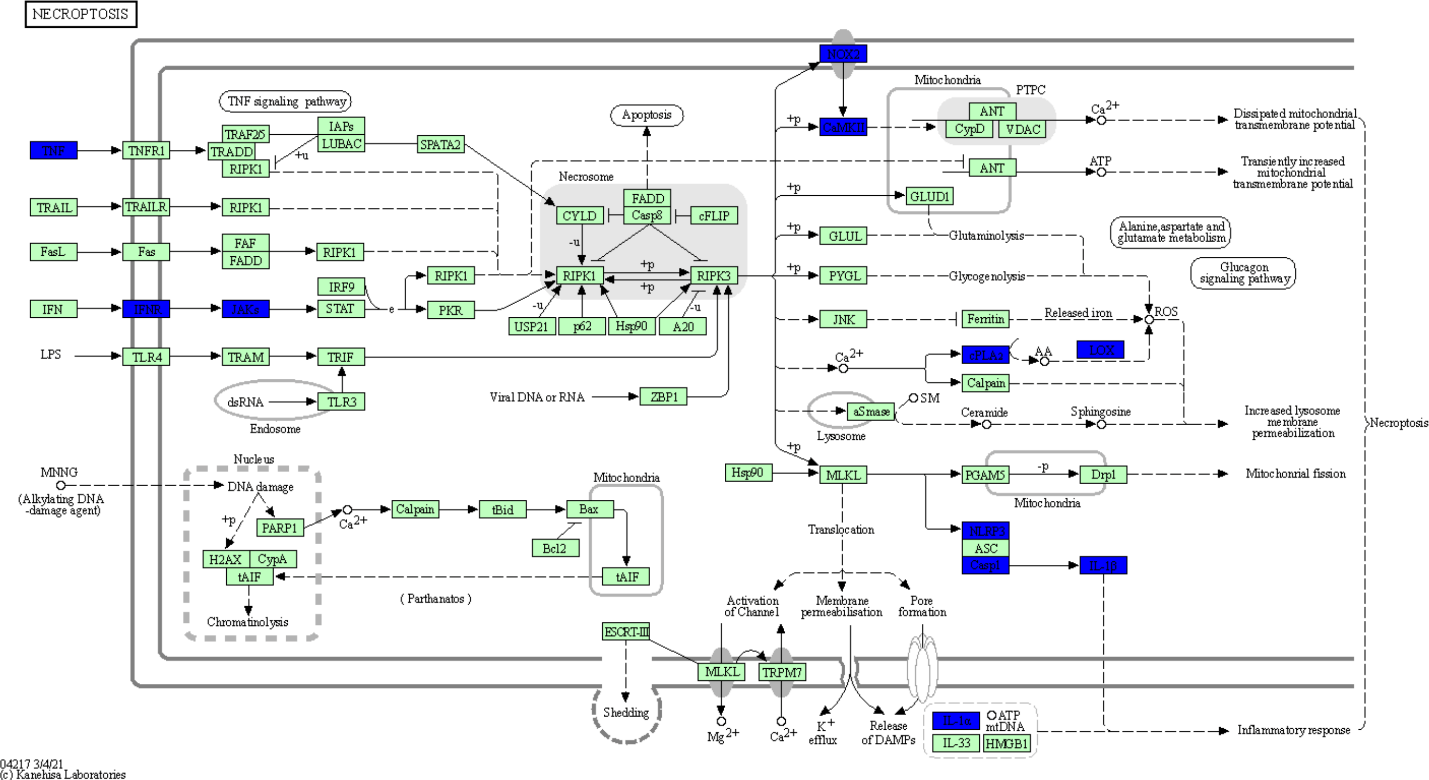
**

**Figure S3 (Cont.)**

**K.** ODC-F

**
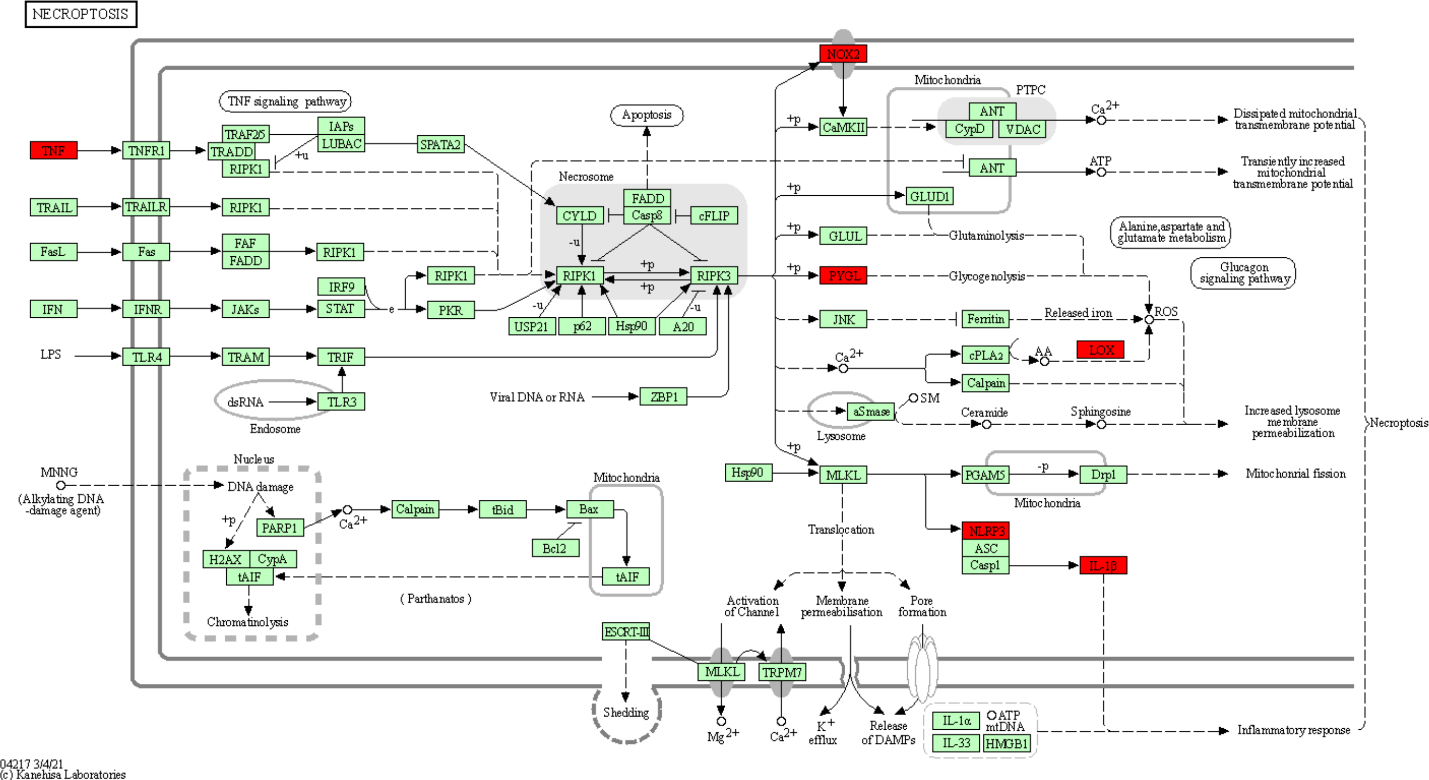
**

**Figure S3.**

Related to Figure 3. KEGG pathway diagrams with hDEGs of the indicated sex and cell type indicated by red or blue. Red genes are those that were significantly increased by fetal hypoxia, and blue are those that were significantly decreased by fetal hypoxia. **A** Male MG hDEGs in the NF-Kappa B signaling pathway. **B** Male MG hDEGs in the TNF signaling pathway. **C** Female NR hDEGs in the Phagosome pathway. **D** Male NR hDEGs in the Phagosome pathway. **E** Female ODC hDEGs in the Phagosome pathway. **F** Female NR hDEGs in the Ferroptosis pathway. **G** Male NR hDEGs in the Ferroptosis pathway. **H** Female ODC hDEGs in the Ferroptosis pathway. **I** Female NR hDEGs in the Necrooptosis pathway. **J** Male NR hDEGs in the Necrooptosis pathway. **K** Female ODC hDEGs in the Necrooptosis pathway.

**Figure S4**


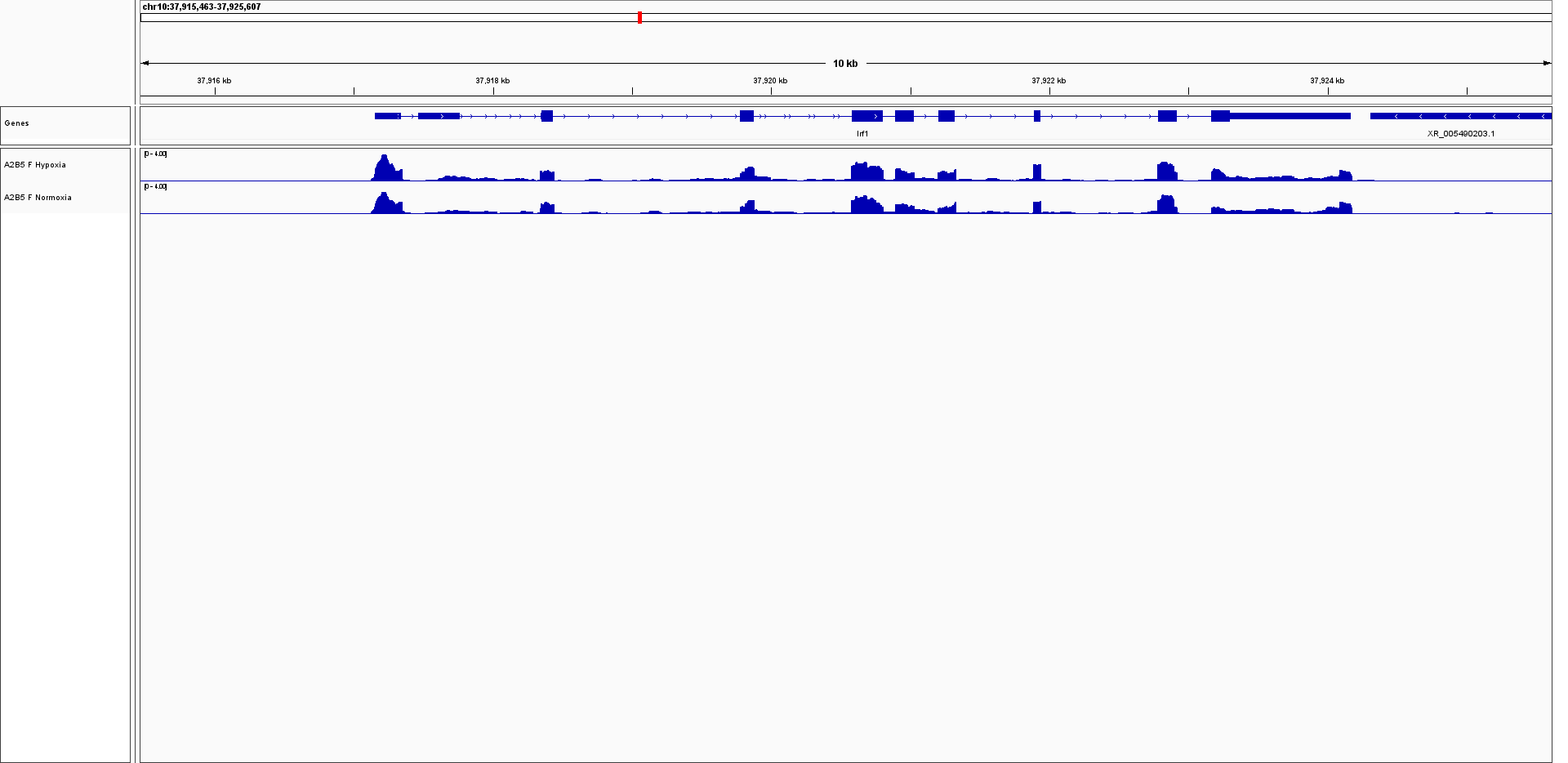
**A. A2B5_IRF1**

**
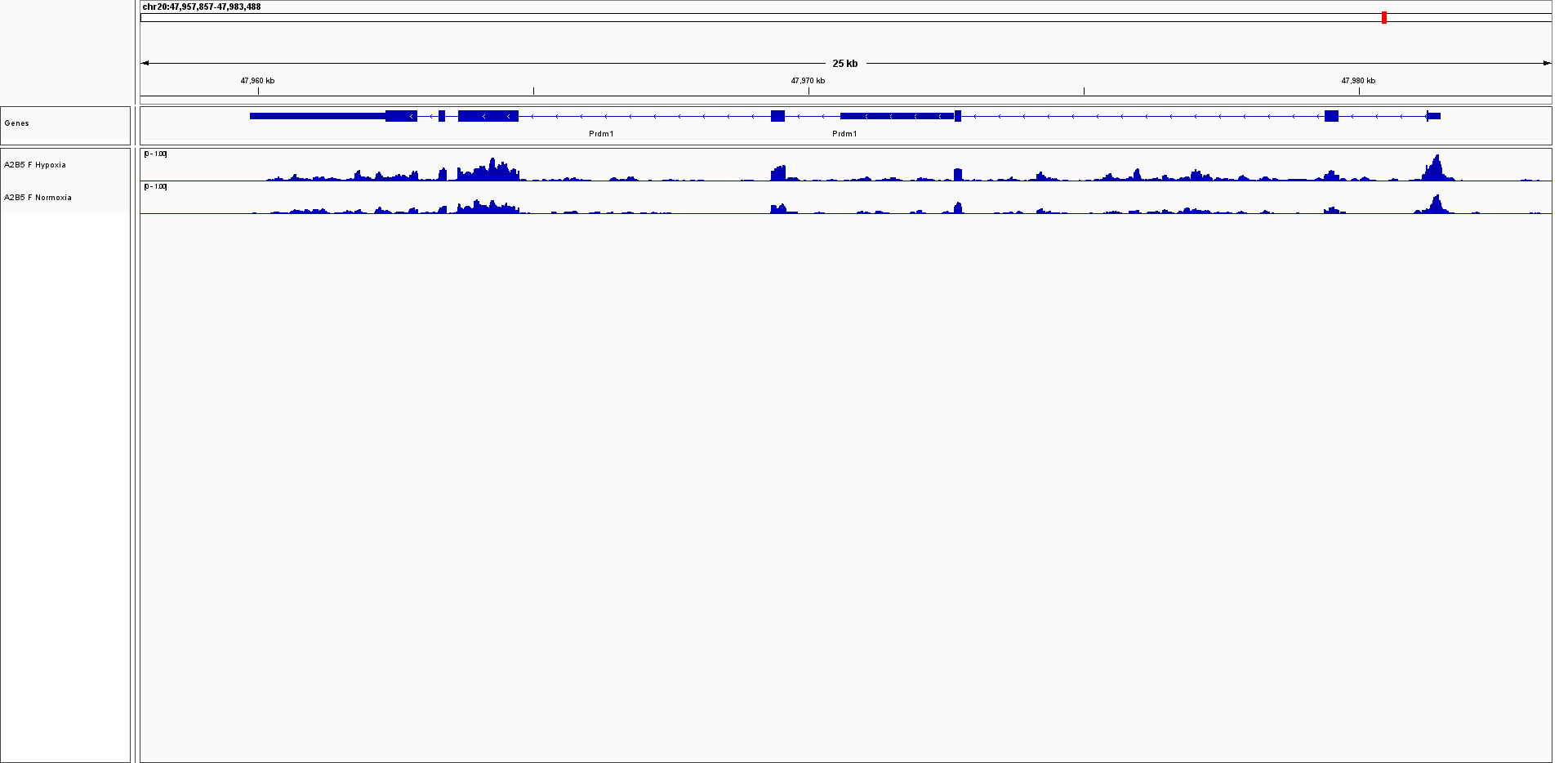
B. A2B5_PRDM1**

**
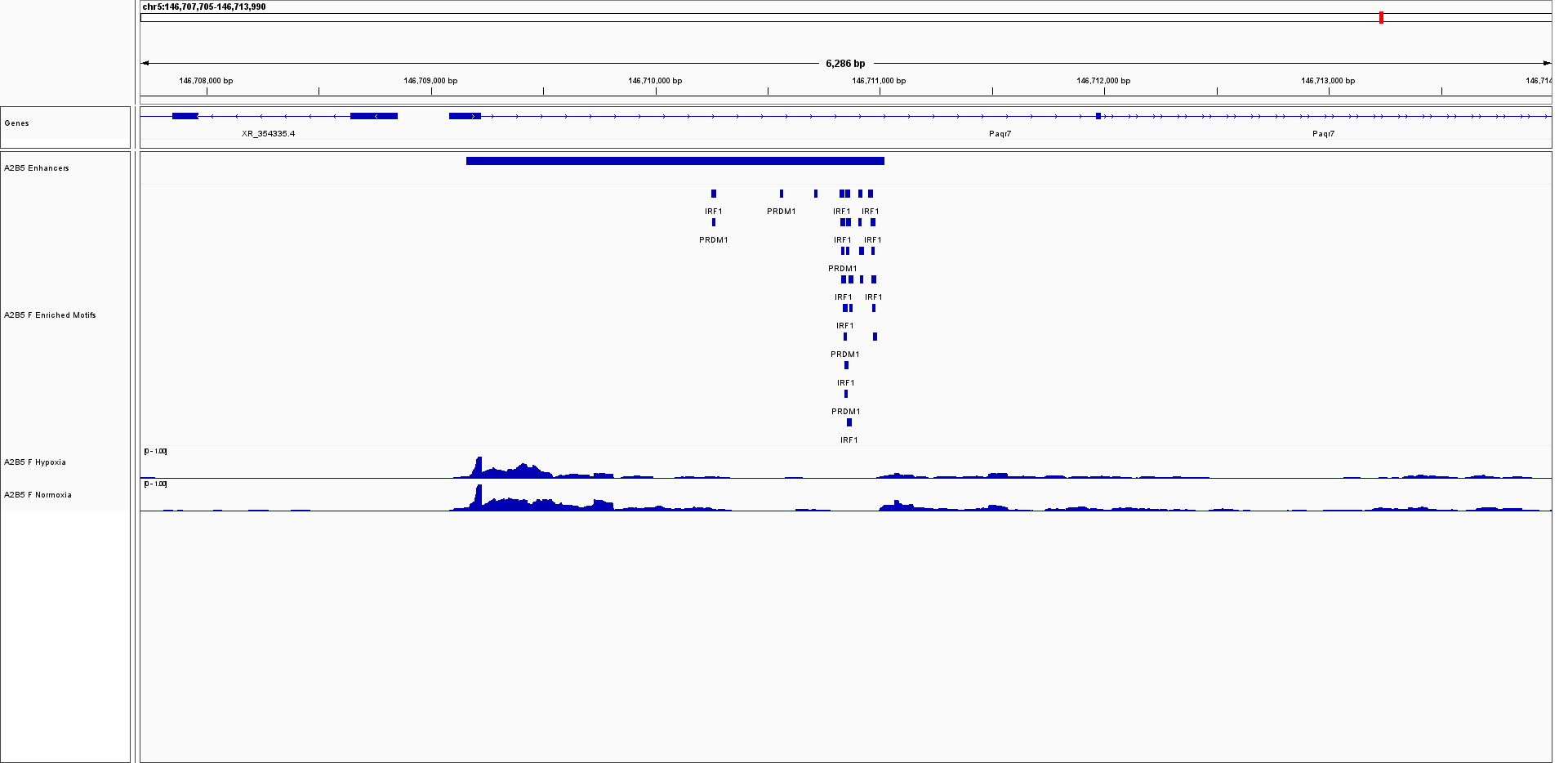
C. A2B5_Motifs**

**Figure S4 (Cont.)**

**D. MG_ZIC1**

**
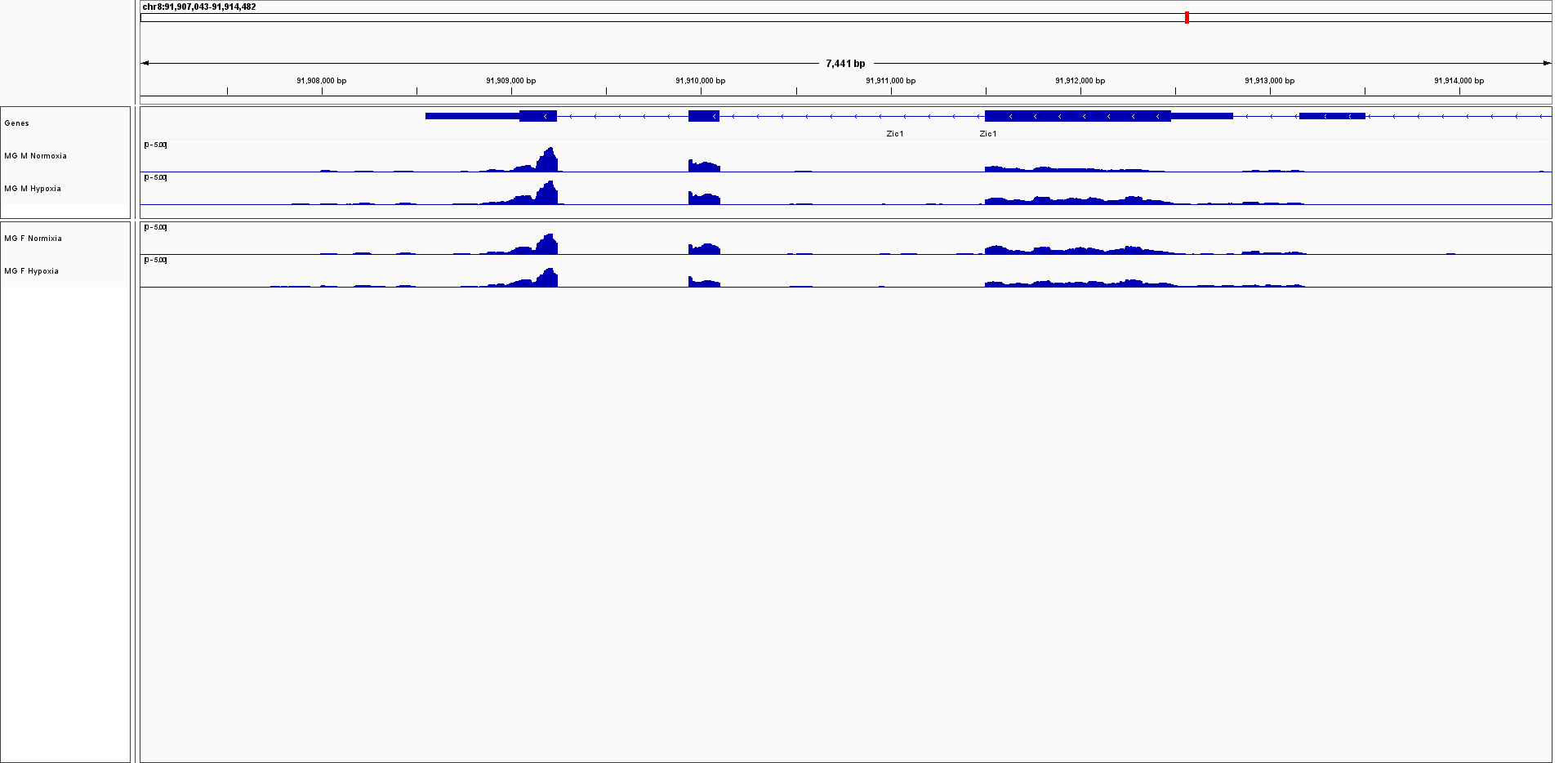
**

**E. MG_DKK2**

**
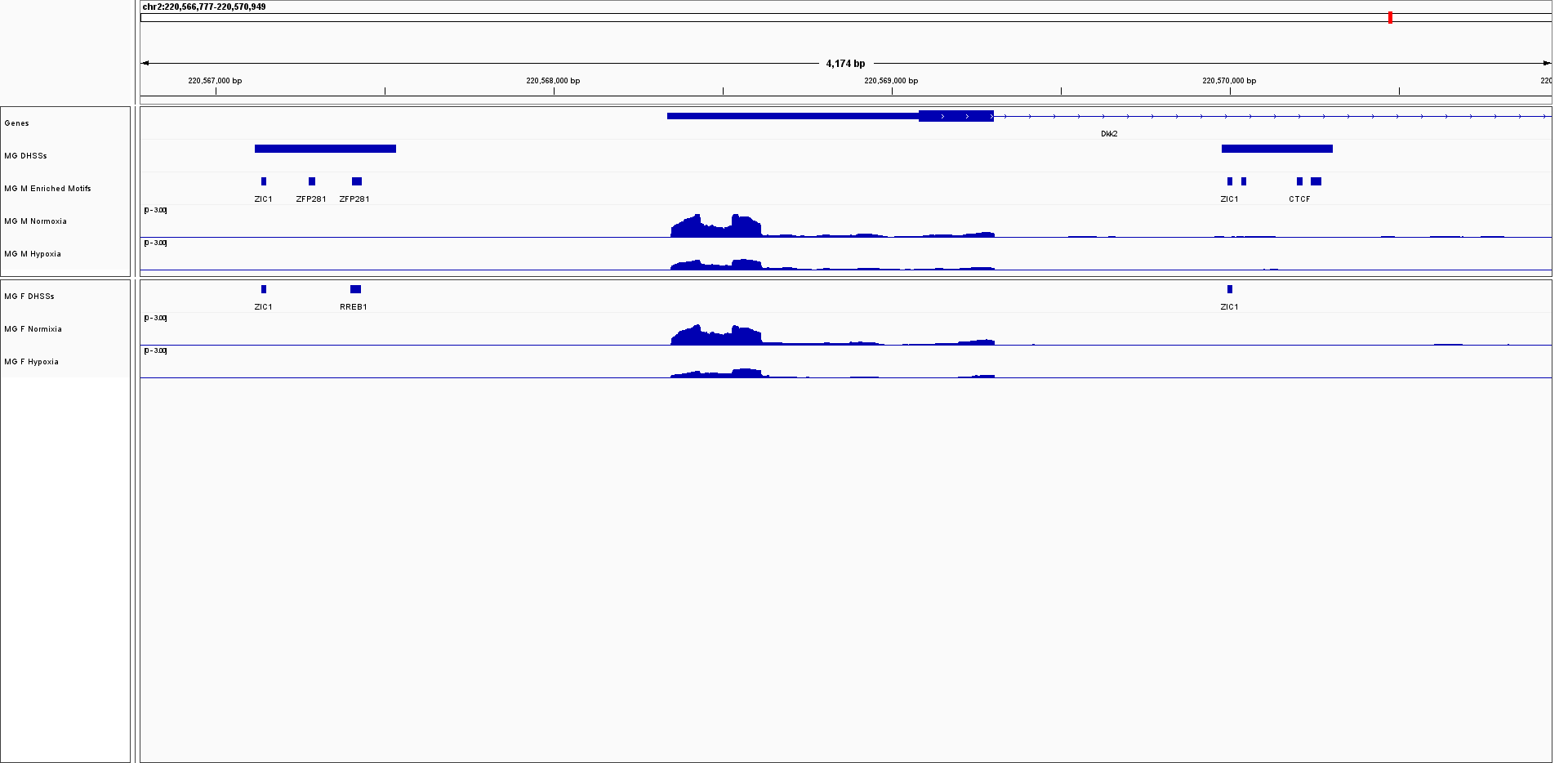
**

**F. MG_LILRB2**

**
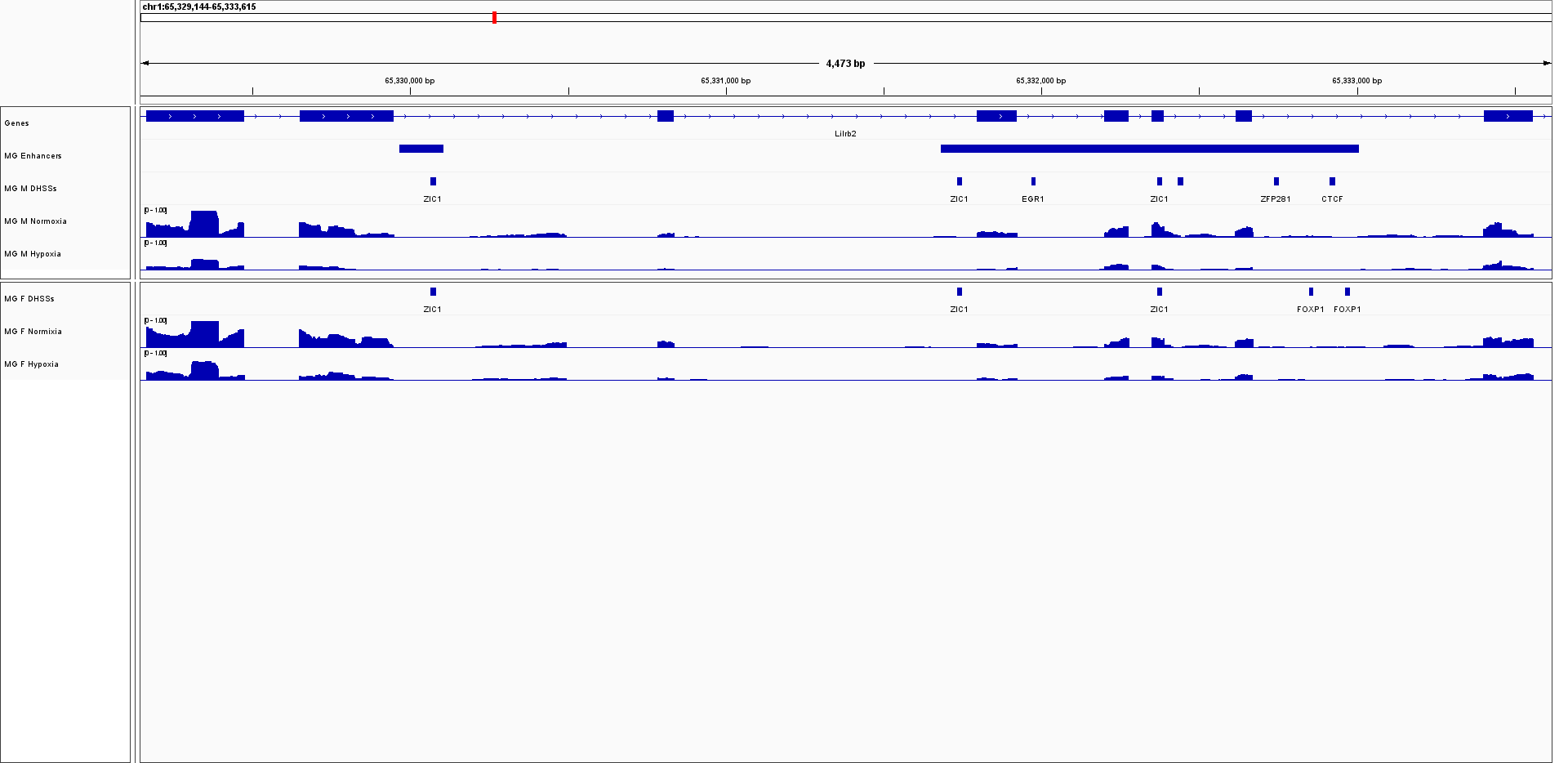
**

**Figure S4 (Cont.)**

**G. NR_HNF1B**

**
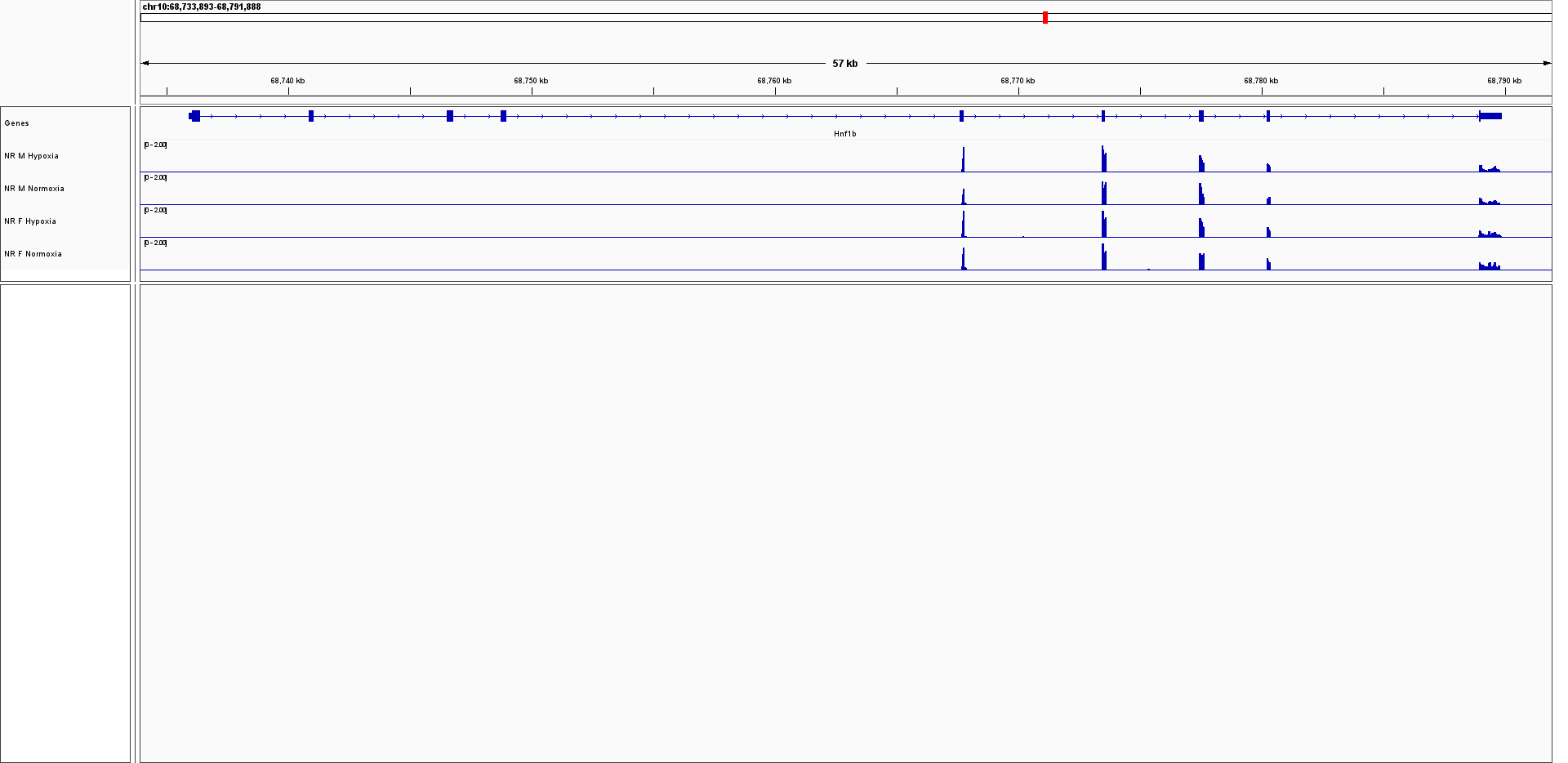
**

**
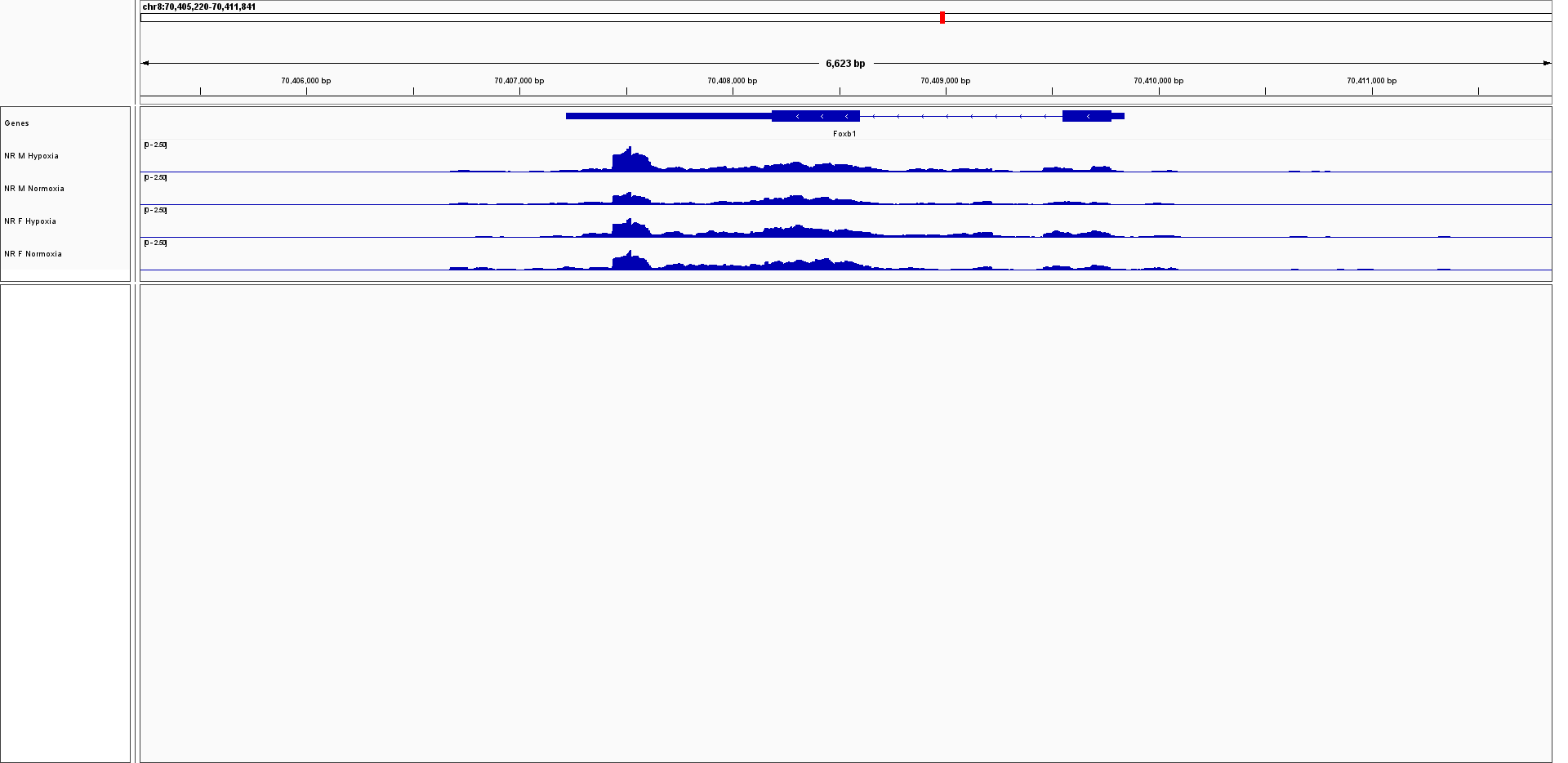
H. NR_FOXB1**

**
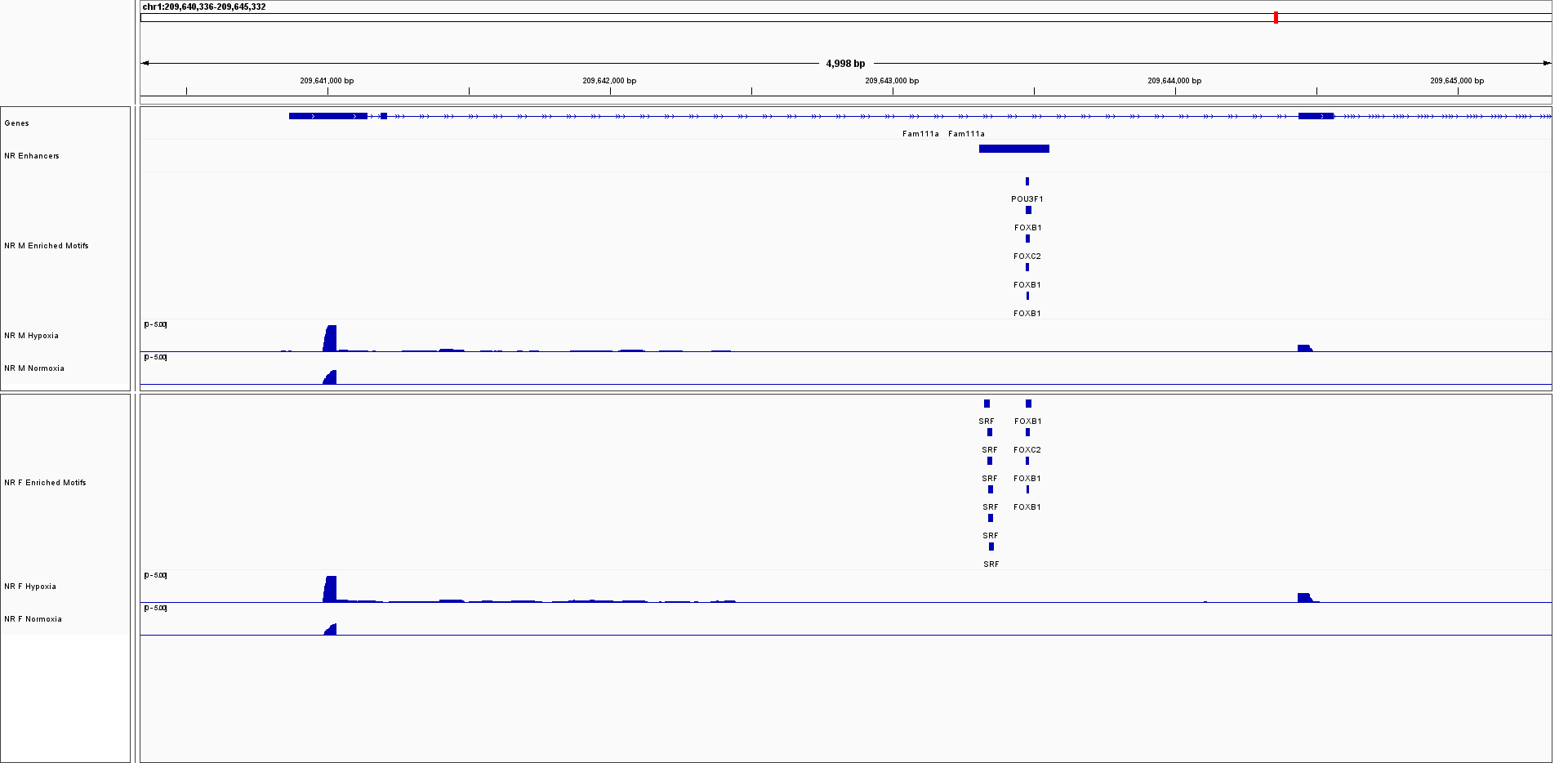
I. NR_Motifs**

**Figure S4 (Cont.)**

**J. ODC_EGR2**

**
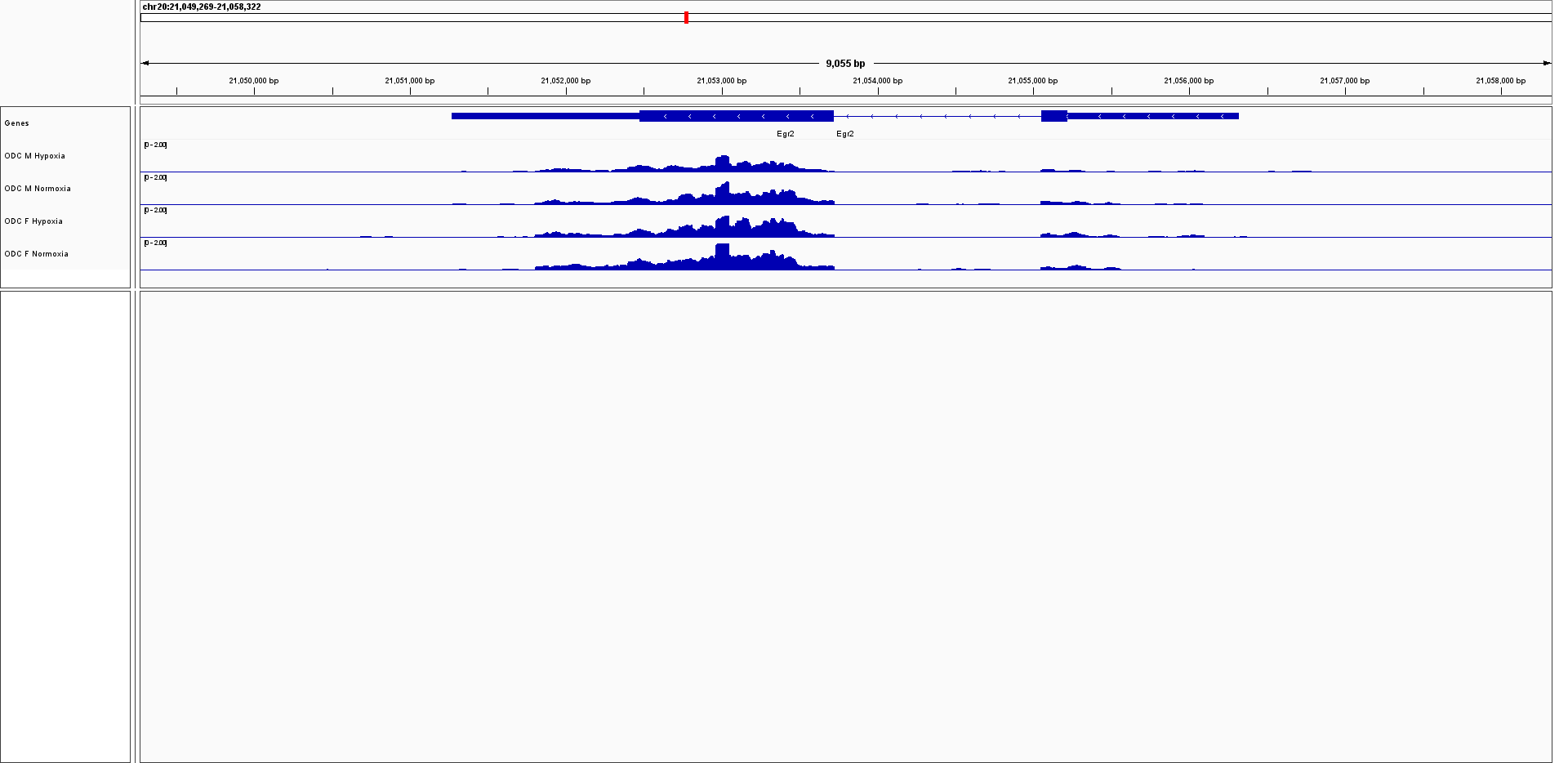
**

**
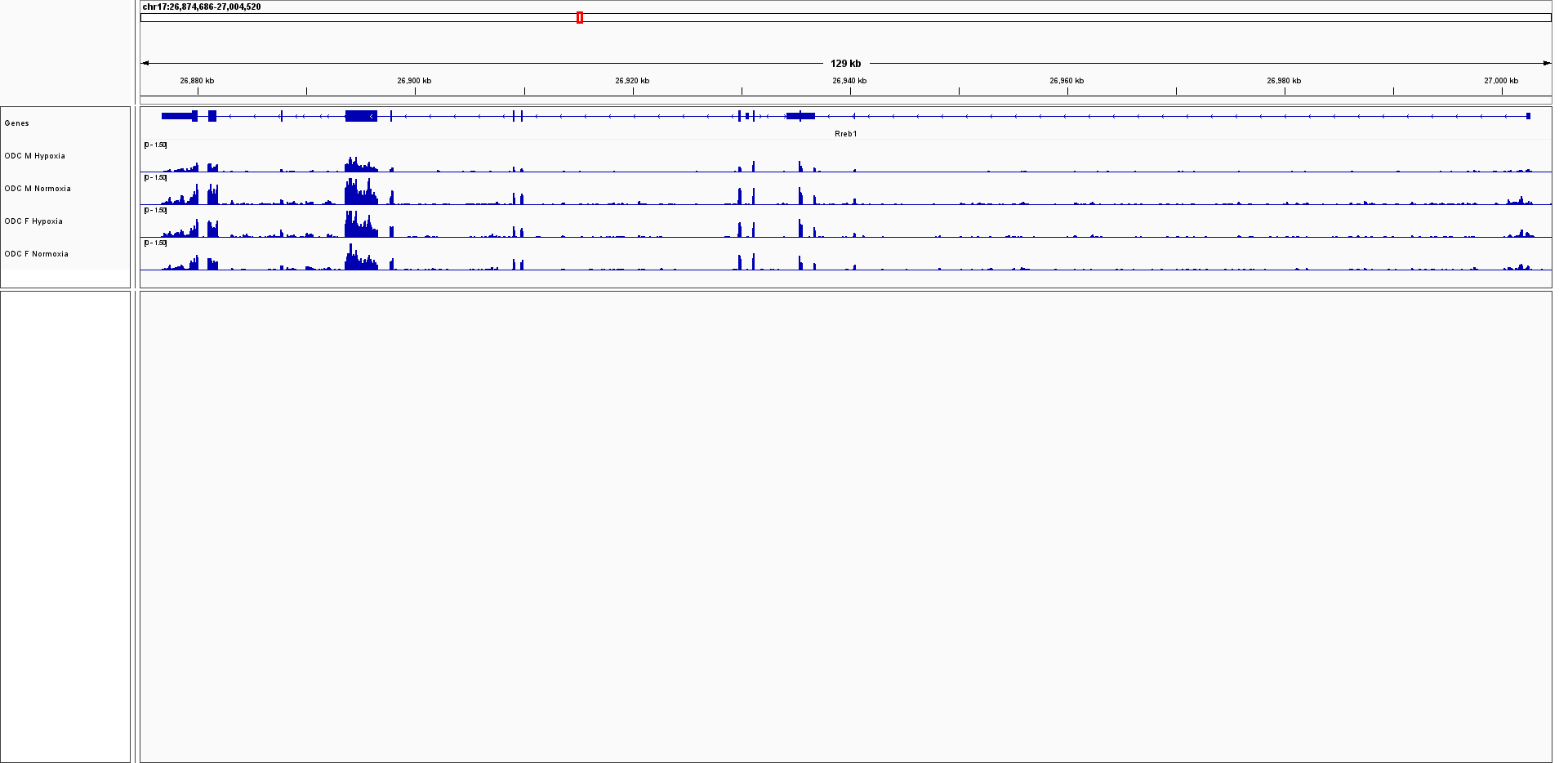
K. ODC_RREB1**

**
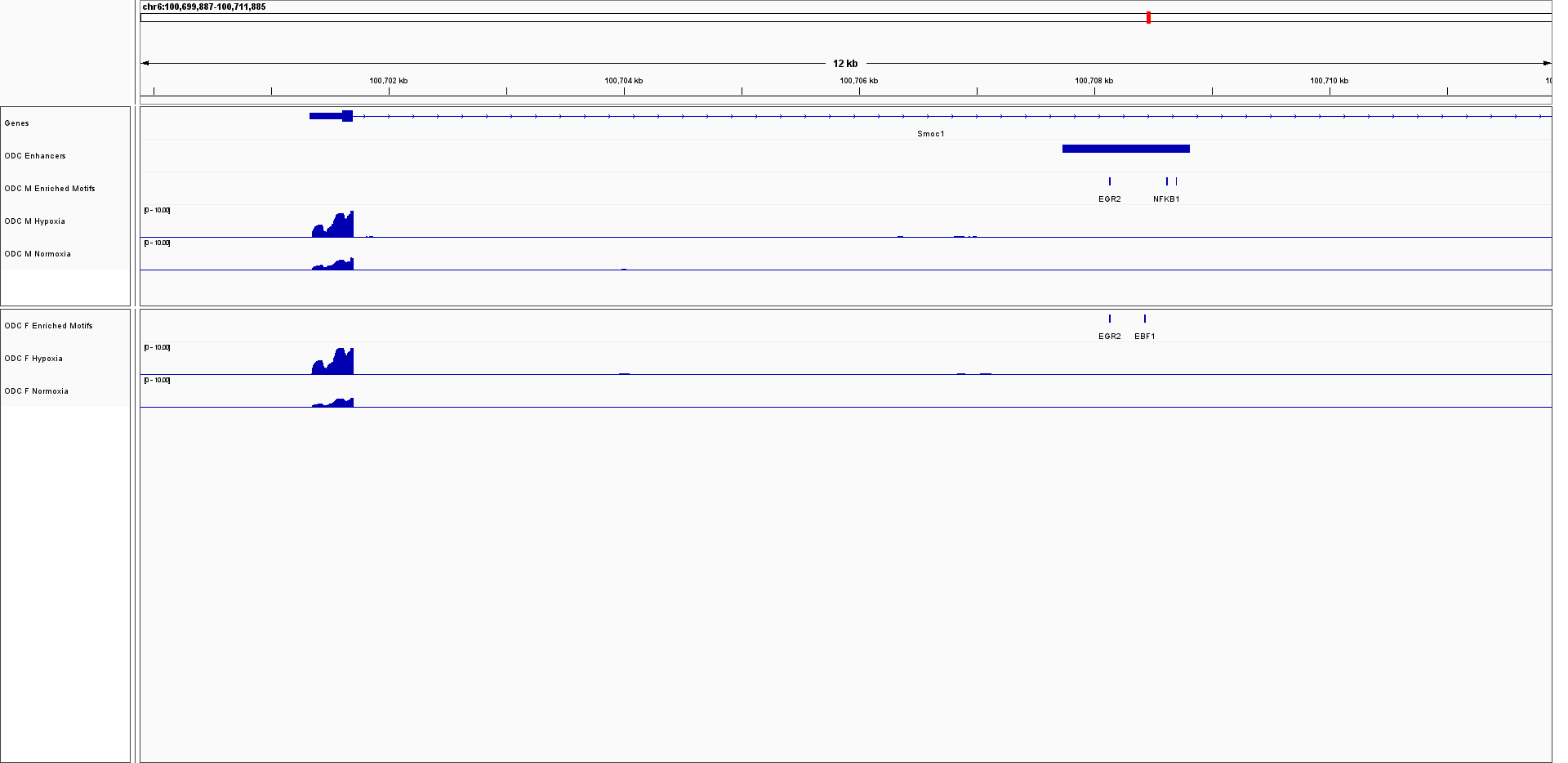
L. ODC_SMOC1**

**Figure S4**

Related to Figure 4. Genome browser views of examples related to Figure 4. Blue signal in tracks represents RPM-normalized expression from RNA-seq, with replicates pooled. Tracks with TF motifs only display motifs that were significantly enriched from the analysis related to Figure 4. **A** A2B5^+^ cell expression of the Irf1 TF. **B** A2B5^+^ cell expression of the Prdm1 TF. **C** Motifs near a female A2B5^+^ cell hDEG. **D** MG expression of the Zic1 TF. **E,F** Motifs near examples of MG hDEGs. **G** NR expression of the Hnf1b TF. **H,I** Motifs near examples of NR hDEGs. **J** ODC expression of the Egr2 TF. **K** ODC expression of the Rreb1 TF. **L** Motifs near an ODC hDEG.
