# Supplementary material for: Fetal hypoxia results in sex- and cell type-specific alterations in neonatal transcription in rat oligodendrocyte precursor cells, microglia, neurons, and oligodendrocytes
Source: Cell Biosci. 2023 Mar 17;13:58. doi: 10.1186/s13578-023-01012-8 (PMC10022003; doi:10.1186/s13578-023-01012-8)
Supplement: Supplementary file 2 — Additional file 2: Table S1. Hypoxia vs. Normoxia DEG (hDEG) counts. Table S2. Male vs. Female DEG counts. Table S3. Interaction DEG counts. Table S4. PCR primer details. [file 13578_2023_1012_MOESM2_ESM.docx]

**Table S1. Hypoxia vs. Normoxia DEG (hDEG) counts.**


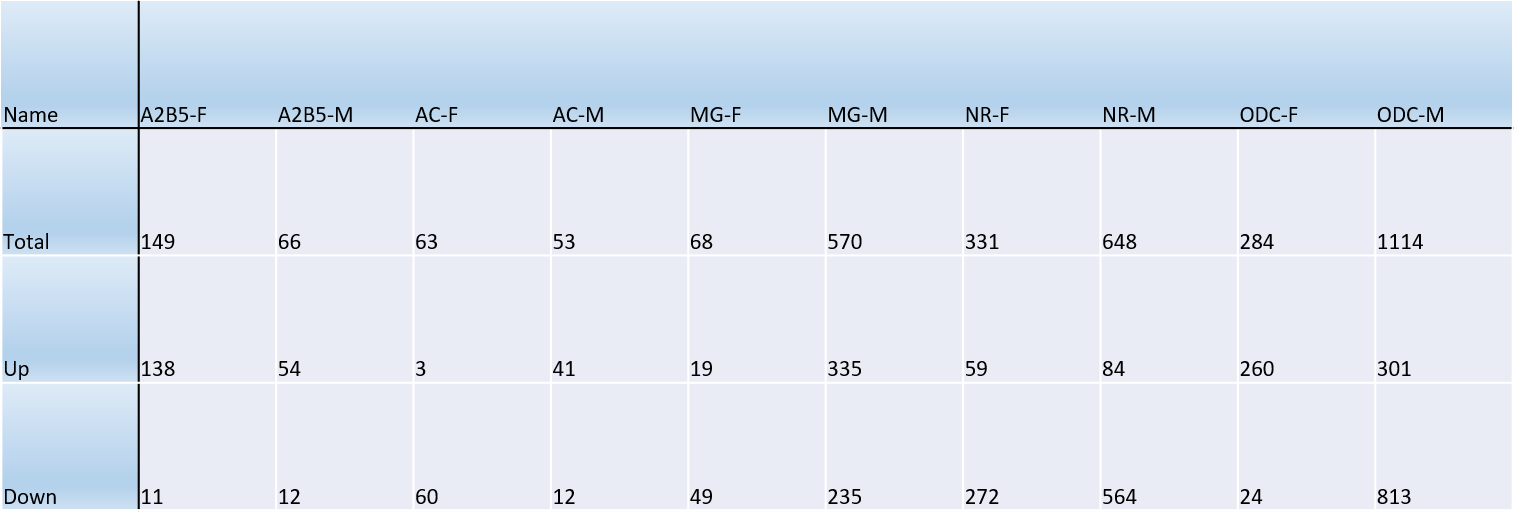


**Table S2. Male vs. Female DEG counts.**


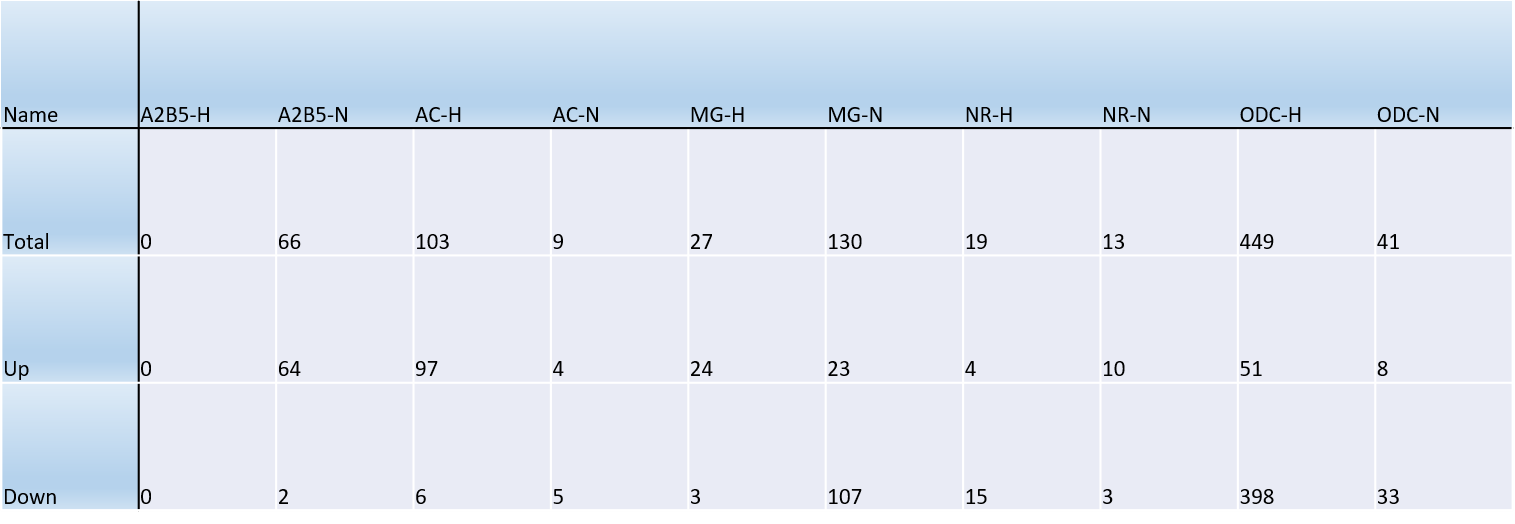


**Table S3. Interaction DEG counts.**


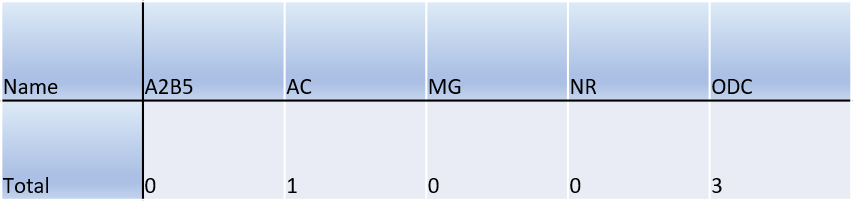


**Table S4. PCR primer details.**

**Abcg2 mRNA (**Rattus norvegicus**)**

Abcg2-F: 5’- CCCGTGGTATCTCTGGAGGA, tm: 60.11

Abcg2-R: 5’- GCTGTGCTTGAGTCCAAACC, tm: 59.69

**Amplicon: 115 bp**

# Alox5 arachidonate 5-lipoxygenase [Rattus norvegicus]

Gene ID: 25290, updated on 24-Apr-2022

# Rattus norvegicus arachidonate 5-lipoxygenase (Alox5), mRNA

Accession Number: NM_012822.2

ALOX5-F: 5’-ATGATGGACTGCTCGTGTGG, Tm: 60.11

ALOX5-R: 5’- TCACGAAGTCCTGCAGTTCC, Tm: 59.97

**Amplicon: 114 bp**

# Alox15 arachidonate 15-lipoxygenase [Rattus norvegicus]

Gene ID: 81639, updated on 24-Apr-2022

# Rattus norvegicus arachidonate 15-lipoxygenase (Alox15), mRNA

Accession: NM_031010.2

**Alox15 QPCR primers (primer pair 5)**

Alox15-F: 5’-ACCTGTGGTTGGTTGGACAG, Tm: 60.11

Alox15-R: 5’-GTGGCCCAAGGTATCCTGAC, Tm: 60.11

**Amplicon: 111 bp**

**Hspa1a mRNA (**Rattus norvegicus**)**

Hspa1a-F: 5’-CGCTCCAGGTGTGATCTAGG, tm: 59.61

Hspa1a-R: 5’-TTGCAGACCGAACGAAGGAG, tm: 60.32

**Amplicon: 92 bp**

**Ikbke**

**Ikbke inhibitor of nuclear factor kappa B kinase subunit epsilon [*Rattus norvegicus* (Norway rat)]**

Gene ID: 363984, updated on 22-May-2022

<https://www.ncbi.nlm.nih.gov/gene/363984>

# Rat inhibitor of nuclear factor kappa B kinase subunit epsilon (Ikbke), mRNA

**NCBI Reference Sequence: NM_001108854.1**

**ACC no: NM_001108854.1**

**[Primer-BLAST](https://www.ncbi.nlm.nih.gov/tools/primer-blast/index.cgi)» JOB ID: dX-qiQQnCY8utZmwlNC9gu7LrLDD2Letwg**

**Ikbke F: 5’-GGTGTTCCAGGAGGAGTGTG, Tm:59.96**

**Ikbke R: 5’-ATGAGGTGCAGGTGGTTCTG, Tm:59.96**

**Amplicon: 102 bp**

# Il1a interleukin 1 alpha [Rattus norvegicus]

Gene ID: 24493, updated on 22-May-2022

# Rattus norvegicus interleukin 1 alpha (Il1a), mRNA

**ACC: NM_017019.2**

IL-1a-F: 5’- AATCCTCTGAGCTTGCCAGG, tm: 59.74

IL-1a-R: 5’- TCCACGGATGTGGGAAACAG, tm: 59.96

**Amplicon: 119 bp**

# Il1b interleukin 1 beta [ Rattus norvegicus]

# Gene ID: 24494, updated on 22-May-2022

# Rattus norvegicus interleukin 1 beta (Il1b), mRNA

Accession: NM_031512.2

**IL-1b QPCR primers (primer pair 5)**

IL1b -F: 5’-CCTCTGTGACTCGTGGGATG, tm: 59.83

IL1b -R: 5’-TCACATGGGTCAGACAGCAC, tm: 59.96

**Amplicon: 117 bp**

**Il-10 interleukin 10 [Rattus norvegicus (Norway rat)]**

Gene ID: 25325, updated on 22-May-2022

<https://www.ncbi.nlm.nih.gov/gene/25325>

**IL-10 mRNA**

<https://www.ncbi.nlm.nih.gov/nuccore/NM_012854.2>

**ACCESSION: NM_012854**

**[Primer-BLAST](https://www.ncbi.nlm.nih.gov/tools/primer-blast/index.cgi)» JOB ID:UFqPox2KECI3HBUZGHkxK2JiIBlPcTsETg**

**IL-10 QPCR primers (5’>3’: 20-mer each**

**IL-10F: 5’-** **CCTGGTAGAAGTGATGCCCC, Tm: 59.82**

**IL-10R: 5’-** **GCAGCTGTATCCAGAGGGTC, Tm: 59.89**

**Amplicon: 101 bp**

# Internal Control

# Actin Beta Rattus norvegicus (Norway rat)

Gene ID: 81822, updated on 24-Apr-2022

**Rat beta actin mRNA**

<https://www.ncbi.nlm.nih.gov/nuccore/NM_031144.3>

**ACCESSION NM_031144**

**Rat beta actin QPCR primer designed 060622**

R-Actin-F: 5’-CCTCACTGTCCACCTTCCAG, Tm: 59.68

R-Actin-R: 5’-GGGTGTAAAACGCAGCTCAG, Tm: 59.68

**Amplicon: 120 bp**

# Lcn2 lipocalin 2 [Rattus norvegicus]

Gene ID: 170496, updated on 24-Apr-2022

# Rattus norvegicus lipocalin 2 (Lcn2), mRNA

**Accession Number: NM_130741.1**

Lcn2-F: 5’- **CCGACACTGACTACGACCAG**, tm: 59.83, %GC: 60

Lcn2-R: 5’- **CCTTGAGGCCCAGAGACTTG**, tm: 60.04, %GC: 60

**Amplicon: 156 bp**

**LOX** Lysyl oxidase [ Rattus norvegicus]

Gene ID: 24914, updated on 24-Apr-2022

**Lox mRNA**

# Rattus norvegicus lysyl oxidase (Lox), mRNA

ACC: NM_017061.2

Lox-F: 5’-TCCTGGCCTCCCATCTACTC, tm: 60.11

Lox-R: 5’- CAGAAAGAGCACGGTCCAGG, tm: 60.67

**Amplicon: 120 bp**

# NfKbib

# NfKb inhibitor beta [Rattus norvegicus (Norway rat)]

Gene ID: 81525, updated on 24-Apr-2022

**<https://www.ncbi.nlm.nih.gov/gene/81525>**

**Nfkbib: mRNA**

# Rattus norvegicus NFKB inhibitor beta (Nfkbib), mRNA

# <https://www.ncbi.nlm.nih.gov/nuccore/NM_030867.2>

**Accession number**: NM_030867.2

**Nfkbib QPCR primer ordered to IDT**

**Nfkbib-F1: 5’-** CCACCCAAGAGATGCCTCAG, Tm: 60.11

**Nfkbib-R1: 5’-** CAGCTCCTCTTCGTTCCCTG, Tm: 60.11

**Amplicon: 118 bp**

**Primer pair 4 from Primer BLAST List**

**Pygl, glycogen phosphorylase L [*Rattus norvegicus*]**

Gene ID: 64035, updated on 22-May-2022

# Rattus norvegicus glycogen phosphorylase L (Pygl), mRNA

ACC: NM_022268.1

PYGL-F: 5’-CGCTCTGGACAAGAAAGGGT, tm: 59.96

PYGL-R: 5’-GAGGTCTGGCTGATTGGGAG, tm: 59.82

**Amplicon: 115 bp**

# Steap3 STEAP3 metallo reductase [Rattus norvegicus]

Gene ID: 170824, updated on 22-May-2022

# Rattus norvegicus STEAP3 metalloreductase (Steap3), mRNA

Accession Number: NM_133314.2

STEP-F: 5’- AACCATCGAAGCCTCCCTTG

STEP-R: 5’- GGCTGGTTCTCTGCCTACAG

**Amplicon: 103 bp**

**TNF tumor necrosis factor, Rattus norvegicus (Norway rat)**

Gene ID: 24835, updated on 22-May-2022

# Rattus norvegicus tumor necrosis factor (Tnf), mRNA

Accession Number: NM_012675.3

TNF-F: 5’-CAGCAACTCCAGAACACCCT, Tm: 59.89

TNF-R: 5’-GGAGGGAGATGTGTTGCCTC, Tm: 60.11

**Amplicon: 105 bp**

# Tnfsf13b TNF superfamily member 13b Rattus norvegicus (Norway rat)

Gene ID: 498666, updated on 22-May-2022

**<https://www.ncbi.nlm.nih.gov/gene/498666>**

# Rattus norvegicus TNF superfamily member 13b (Tnfsf13b), mRNA

# <https://www.ncbi.nlm.nih.gov/nuccore/NM_001395721.1>

# Accession number: NM_001395721

Tnfsf13b **QPCR primer ordered to IDT**

**Tnf-F1: 5’-** CACTGCCCAACAATTCCTGC, Tm: 60.04

**Tnf -R1: 5’-** TCCGTTCCGTGAAATCTGGG, Tm: 60.04

# **Amplicon: 107 bp**
